# The effect of fructose exposure on amino acid metabolism among Chinese community residents and its possible multi-omics mechanisms

**DOI:** 10.1038/s41598-023-50069-5

**Published:** 2023-12-19

**Authors:** Ouyan Rang, Xinru Qin, Yonghong Tang, Lin Cao, Guojuan Li, Xiaocheng Liu, Jing Zhong, Mu Wang

**Affiliations:** 1 Clinical Mass Spectrometry Laboratory of Clinical Research Institute and Department of Basic Medicine of Nuclear Industrial Hygiene School, Affiliated Nanhua Hospital, Hengyang Medical School, University of South China, Hengyang, Hunan 421001 People’s Republic of China; 2 School of Public Health, University of South China, Hengyang, Hunan 421001 People’s Republic of China; 3 The First Affiliated Hospital, Hengyang Medical School, Institute of Clinical Medicine, Cancer Research Institute, University of South China, Hengyang, Hunan 421001 People’s Republic of China

**Keywords:** Systems biology, Biomarkers

## Abstract

The consumption of fructose has increased dramaticly during the last few decades, inducing a great increase in the risk of intrahepatic lipid accumulation, hypertriglyceridemia, hyperuricemia and cancer. However, the underlying mechanism has not yet been fully elucidated. Amino acid metabolism may play an important role in the process of the diseases caused by fructose, but there is still a lack of corresponding evidence. In present study, we provide an evidence of how fructose affects amino acids metabolism in 1895 ordinary residents in Chinese community using UPLC-QqQMS based amino acid targeted metabolomics and the underlying mechanism of fructose exposure how interferes with amino acid metabolism related genes and acetylated modification of proteome in the liver of rats model. We found people with high fructose exposure had higher levels of Asa, EtN, Asp, and Glu, and lower levels of 1MHis, PEtN, Arg, Gln, GABA, Aad, Hyl and Cys. The further mechanism study displayed amino acid metabolic genes of ***Aspa*, *Cndp1*, *Dbt*, *Dmgdh,*** and toxic metabolites such as *N*-acetylethanolamines accumulation, interference of urea cycle, as well as acetylated modification of key enzymes in glutamine metabolic network and glutamine derived NEAAs synthesis pathway in liver may play important roles in fructose caused reprogramming in amino acid metabolism. This research provides novel insights of the mechanism of amino acid metabolic disorder caused by fructose and supplies new targets for clinical therapy.

## Introduction

The consumption of fructose has increased rapidly since the introduction of high fructose corn syrup (HFCS) in 1967^1^. HFCS is a product fermented from corn, and usually has a composition of 42% fructose (HFCS-42) or 55% fructose (HFCS-55)^2^, which has several commercial advantages over crystalline sugar (sucrose), such as convenience to transport and use, less dependence on foreign sources^2^, and possibly being sweeter than other sugar^2,3^. These properties made the consumption of HFCS-containing beverages dramatically increase during the past several decades all over the world.

Based on the United States Department of Agriculture (USDA) records, the average fructose intake continuously increased from 37 to 49 g/day between 1977 and 2004^4^. According to the International Sugar Organization reports, the average per capita sugar consumption progressively increased from 56 to 65 g/day in the 1986–2007 period^5^. Within the Dutch National Food Consumption Survey 2007–2010, median fructose intake was 46 g/day in the Dutch population aged 7–69 years^6^. Data from China National Nutrition and Health Surveillance showed the average dietary fructose intake of Chinese residents aged 45 and above was 8.29 g/day^7^. Although existing data suggested that the intake of fructose by Chinese residents was slightly lower than that of residents in European and American countries, fructose exposure has already caused great concern for the health risk of Chinese residents as the food patterns and diet have greatly changed accompanied by the expanding economy during the last decades in China.

Fructose can cause series of health damage, such as increasing risks of obesity, dyslipidemia, hypertension and cardiometabolic syndrome^3,8–10^. Recently, multiple studies have demonstrated that fructose can act as an additional fuel for cancer cells, which provides important supports for the survival, proliferation and metastasis of cancer cells^11,12^. These data indicated that fructose was associated with almost all serious human diseases. However, the mechanism has not been fully elucidated yet.

It is well known that amino acids (AAs) are not only cell signaling molecules but are also regulators of gene expression and key precursors for syntheses of hormones and low-molecular weight nitrogenous substances with each having enormous biological importance^13^. And an optimal balance among AAs in the circulation was undoubtedly crucial for whole body health status. Previous study reported fructose could reprogram cellular metabolic pathways to favour glutaminolysis and oxidative metabolism, which are required to support increased inflammatory cytokine production in immune cells^14^. However, little is currently known whether fructose exposure can cause disorders of amino acid metabolism at the level of large scale cross-sectional population research.

Here, we provide an evidence of how fructose affects amino acids metabolism in 1895 ordinary residents in Chinese community using UPLC-QqQMS based amino acid targeted metabolomics and the underlying mechanism of fructose exposure how interferes with amino acid metabolism related genes and acetylated modification of proteome in the liver of animals model. Taken population and animal based studies together, this work demonstrates that fructose exposure significantly changed amino acid metabolism in the population level and the mechanism may involve amino acid metabolic genes of ***Aspa***, ***Cndp1***, ***Dbt***, ***Dmgdh***, and toxic metabolites such as *N*-acetylethanolamines accumulation, interference of urea cycle, as well as acetylation modification of key enzymes in glutamine derived NEAAs synthesis pathway in liver.

## Materials and methods

### Materials and equipment

Methanol and acetonitrile were purchased from Merck, China. Acquity UPLC HSS T3 column (1.8 μm, 2.1 mm * 50 mm) and ultra performance liquid chromatography and tandem mass spectrometry (TQSm) were purchased from waters, USA. Derivatizing agent (6-Amino-quinolyl-*N*-hydroxysuccinimidylcarbamate), and amino acid standards such as His, 1MHis, 3MHis, Hyp, Asn, PEtN, Arg, Car, Ans, Asa, Ser, Tau, Gln, EtN, Gly, Sar, bAla, Thr, Asp, Glu, Cit, Ala, GABA, bAib, Pro, aAd, Hyl, Hcit, Abu, Val, Met, Tyr, Cth, Cys, Leu, Ile, Phe, Trp and Lys were perchased from Sigma-Aldrich, USA. Internal standard products (Pro-^13^C_5_^15^N, His-^13^C_6_, Arg-^13^C_6_, EtN-^13^C_2_, Gly-d_2_, Sar-d_3_, Ala-d_3_, Ser-^13^C_3_, Tau-d_4_, Thr-^13^C_2_, Asn-^13^C_4_^15^N_2_, Asp-d_3_, PEtN-d_4_, Glu-^13^C_5_, Gln-^13^C_5_, Met-d_3_, aAd-d_6_, Cth-d_4_, Tyr-^13^C_9_^15^N, GABA-d_6_, Abu-d_3_, Ile-d_10_, Lys-d_8_, Glu-^13^C_5_, Phe-d_7_, Cit-d_7_, Val-d_8_, Trp-d_5_, bAla-d_4_, Leu-d_3_, bAib-d_3_) were purchase from Cambridge Isotope Laboratories, England. Real time fluorescence quantitative PCR analyzer (QIAquant96 2 plex), RT2 First Strand Kit (330401), RT2 SYBR Green Fluor qPCR Mastermix (330513), RT2 Profiler PCR Array (PARN-130ZF), RNeasy Mini Kit (74104) and RNase-Free DNase Set (79254) were purchased from QIAGEN, Germany. ZenoTOF 7600 high resolution MS was purchased from SCIEX, Canada. Chromatographic column (Kinetex F5, 2.1*150 mm, 2.6 µm, 00F-4723-AN) was purchased from Phenomenex, USA. Easy nLC chromato- graphic system, Q Exactive mass spectrometer, Micro-ultraviolet photometer, C18 loading column (Acclaim PepMap100, 100 μm * 2 cm), and nanoViper C18, EASY column (10 cm, ID 75 μm, 3 μm, C18-A2) were purchased from Thermo Scientific, USA. SDS (161‐0302), Urea (161‐0731), Dithiothreitol (DTT, 161‐0404), Iodoacetamide (IAA, 163‐2109) were purchased from Bio‐Rad, USA.

### Clinical samples

A total of 1895 permanent residents, who were > 18 (69.04 ± 9.98) years old, from a community of Hengyang City in southern Hunan, China, were recruited. All participants provided written informed consent and completed an individual questionnaire at the time of physical examination for this study. The demographic characteristics of these individuals are recorded in Table 1. A total of 1895 serum samples from the subjects were collected at each visit and were frozen at − 80 °C until analysis. A minimized freeze–thaw cycle was assured to reduce introduced interference as much as possible. The research protocol was approved by the Ethics Committees of the Affiliated Nanhua Hospital, University of South China (Ethical approval number: 2022-KY-167). All experiments were performed in accordance with principles expressed in the Declaration of Helsinki or other relevant guidelines and regulations.

**Table 1:** Basic demography characteristics of 1895 community residents.

	Characteristics
Age	69.04 ± 9.98^a^
Gender (Male/Female)	796/1099
History of diabetes (n)	352
Uric acid (μmol/L)	324.45 ± 78.41^a^
Height (cm)	158.41 ± 8.67^a^
Weight (Kg)	59.33 ± 10.66^a^
Waist circumference (cm)	81.69 ± 10.27^a^
BMI (kg/m^2^)	23.54 ± 3.28^a^
Left systolic blood pressure (mm/Hg)	138.24 ± 19.21^a^
Left diastolic blood pressure (mm/Hg)	73.65 ± 8.24^a^
Right systolic blood pressure (mm/Hg)	141.62 ± 19.19^a^
Right diastolic blood pressure (mm/Hg)	77.10 ± 8.19^a^
Hemoglobin (g/L)	132.58 ± 14.41^a^
White blood cells (10^9^/L)	6.97 ± 10.26^a^
Platelet (10^9^/L)	207.49 ± 76.29^a^
Fasting blood glucose (mmol/L)	6.59 ± 3.87^a^
Serum ALT (U/L)	23.00 ± 17.61^a^
Serum AST (U/L)	23.08 ± 12.99^a^
Total bilirubin (μmol/L)	13.83 ± 4.86^a^
Serum creatinine (μmol/L)	76.44 ± 34.53^a^
Blood urea nitrogen (mmol/ L)	5.57 ± 1.56^a^
Total cholesterol (mmol/L)	4.60 ± 0.96^a^
Triglycerides (mmol/L)	1.51 ± 1.13^a^
Low density lipoprotein cholesterol (mmol/L)	2.52 ± 0.65^a^
High density lipoprotein cholesterol (mmol/L)	1.49 ± 0.32^a^

^a^Values are presented as mean ± standard deviation.

### Amino acid targeted metabolomics analysis

Amino acid targeted metabolomics detection was performed on UPLC-QqQMS platform (waters TQSm). Mobile phase A, water (containing 0.1% formic acid); mobile phase B, acetonitrile; and the flow rate was 0.5 mL/min. The gradient conditions of the mobile phase were as follows: 0 min for A/B 98:2 (v/v), 4 min for A/B 78:22 (v/v), 4.1 min for A/B 5:95 (v/v), 4.4 min for A/B 0:100 (v/v), 4.6 min for A/B 98:2 (v/v), 6 min for A/B 98:2 (v/v). The temperatures of the column and autosampler were maintained at 45 °C and 4 °C, respectively. The injection volume is 2 μL. Triple quadrupole scans were operating in a positive ion mode. The ESI source temperature was 350 °C and the voltage was 3000 V. The scaning detection of ion pairs were depended on the optimized declustering potential and collision energy. Supplementary table 1 showed multiple reaction monitoring (MRM) parameters including parent ions, daughter ions, dwell time, cone voltage and collision voltage of 39 amino acids. Each amino acid was validated through corresponding isotope internal standards. The chromatographic peaks were corrected manually to ensure the accuracy of quantitative determination according to suitable calibration curve (*r*^2^ > 0.99).

Data in every group were expressed with Mean ± standard. Kruskal–Wallis test that was mean comparison of two samples was used to compare among groups^15^. Benjamin Hochberg correction was introduced in this study to reduce type II error rates and to improve the statistical efficiency.

### Serum fructose detection

Serum fructose was detected using microplate method (kit manufacturer: Shanghai Zhenke Biotechnology Co., Ltd.; product number: G20221010H). In brief, fructose in 20 µL serum was converted into glucose by the action of specific enzymes and then the glucose could increase the amount of NADPH under the action of hexokinase enzyme complexes. The content of fructose could be calculated by using the microplate reader to detect the increase of the NADPH at 340 nm.

### Animals and sample collection

Twelve three-week-old male SD rats [Purchased from Hunan SJA Laboratory Animal Co.; Ltd, Changsha, China; Licence: SYXK(Hunan)-2019-0014] were maintained in temperature- and light-controlled (14 h/10 h light/dark cycle) conditions at the Laboratory Animal Center of School of department of zoology, University of South China, a facility approved by the association for assessment and accreditation of laboratory animal care [Licence: SYXK(Hunan)-2020-0002]. The animals were acclimated to the laboratory for 1 week prior to the start of the experiments and were randomized into 2 groups: control group, fructose exposing group (n = 6 rats/group). Animals in each group had free access to food and water. In fructose exposing group, the drinking water was replaced with 5% fructose water for 6 months (the dissolution ratio is: 5 g fructose in 100 mL water, freshly prepared every day). Two groups of rats were provided equal amounts of food with the same ingredients. And rats of fructose exposing group were provided the same volume fructose water as the control group daily.

At the end of the experiment, femoral artery blood was collected into 1.5 mL centrifugal tube after anaesthesia with 3–5% isoflurane. Blood samples were centrifuged for 10 min at 3000 rpm and 4 °C. Serum was collected into microtubes and stored at − 80 °C. All rats were euthanized by cervical dislocation, and the left lateral lobe liver of the rats of every group were separated and were cut into small pieces and mixed evenly, and then grinded into powder in pulverizer after a few minutes for liquid nitrogen freezing. These liver samples were stored at − 80 °C.

All procedures performed in this study were in accordance with ARRIVE guidelines and the ethical standards of the Ethics Committees of Affiliated Nanhua Hospital, University of South China (Ethical approval number: 2021-ky-173).

### Rats liver PCR Array analysis

We applied Rat Amino Acid Metabolism II RT^2^ Profiler PCR Array to profile the expression of 84 key genes important in amino acid biosynthesis and degradation. Mature RNA was isolated using an RNA extraction kit according to the manufacturer's instructions. RNA quality was determined using a spectrophotometer and was reverse transcribed using a cDNA conversion kit. The cDNA was used on the real-time RT^2^ Profiler PCR Array (QIAGEN, Cat. no. PARN-130Z) in combination with RT^2^ SYBR® Green qPCR Mastermix (Cat. no. 330529).

In brief, total RNA was isolated from 50 mg of liver tissue and the isolated RNA was quantified by measuring the absorbance at 260 nm. RNA purity was assessed by the 260/280 nm ratio, and the integrity was checked using 1% agarose gel. Reverse transcription of RNA to cDNA was carried out using Quantiscript Reverse Transcriptase and RT Primer Mix. Reverse transcription reaction mixture (20 µL) contained 14 µL of total RNA, 4 µL of buffer, 1 µL of RT Primer Mix, and 1 µL of Quantiscript Reverse Transcriptase. The resulting mixture was initially incubated at 42 °C for 15 min, and then heating the mixture to 95 °C for 5 min to inactivate the reverse transcriptase. cDNA obtained was stored at − 80 °C until further use.

The final reaction mixture (2000 µL) contained 1000 μL of 2 × QuantiNova SYBR Green PCR Master Mix, 100 μL of cDNA, 900 μL of H_2_O. Before PCR Array test, add 20 µL of the final reaction mixture to each hole of the 96 well plate and the qRT-PCR program consisted of the following temperature profile: initial activation of DNA polymerase at 95 °C for 2 min, and then 45 cycles of denaturation at 95 °C for 5 s and annealing at 60 °C for 10 s.

C_T_ values were exported to an Excel file to create a table of C_T_ values. This table was then uploaded on to the data analysis web portal at http://www.qiagen.com/geneglobe. Samples were assigned to controls and fructose exposing group. The data analysis web portal calculated fold regulation using delta delta C_T_ method, in which delta C_T_ was calculated between gene of interest (GOI) and an average of reference genes (HKG), followed by delta-delta C_T_ calculations (delta C_T_ (Test Group)-delta C_T_ (Control Group)). Fold Regulation was then calculated using 2^ (−delta delta C_T_) formula.

### Rats liver non-targeted metabolomics analysis

#### Liver metabolites extraction

50 ± 5 mg of liver tissue sample was accurately separated and was added 200 μL ultrapure water for homogenization. Added 800 μL methanol: acetonitrile (v:v = 1:1) solution to the homogenate, and vortexed for 30 s, ultrasounded for 10 min (ice bath). The mixed solution was stored in a refrigerator at − 20 °C for 1 h and then was centrifuged at 12,000 rpm (4 °C) for 15 min. The supernatant was blew dry with N_2_. The powder was redissolved with 100 μL acetonitrile: water (v:v = 1:1) solution, and vortexed for 30 s, ultrasounded for 5 min (ice bath). And then the mixed solution was centrifuged at 12,000 rpm (4 °C) for 15 min. The supernatant was injected into the bottle for MS analysis.

#### UPLC-MS detection

Nontargeted metabolomics analysis was performed on SCIEX ZenoTOF 7600 platform. The specific conditions for liquid chromatography were as follows:

Column: Phenomenex Kinetex F5, 2.1*150 mm, 2.6 µm, 00F-4723-AN; Mobile phase A: 0.1% Formic Acid in water; Mobile phase B: 0.1% Formic Acid in acetonitrile; Flow: 200 μL/min; Column temperature: 30 °C; Injection volume: 1 μL. The gradient conditions of the mobile phase were: 0 min for A/B 100:0 (v/v), 2.10 min for A/B 100:0 (v/v), 14.00 min for A/B 5:95 (v/v), 16.00 min for A/B 5:95 (v/v), 16.10 min for A/B 100:0 (v/v), 20 min for A/B 100:0 (v/v).

The specific conditions for mass spectrum were as follows:

Ion Source: TurboIonSpray; Curtain gas: 35; CAD gas: 9; Ion source gas 1 (psi): 55; Ion source gas 2 (psi): 55; Temperature (°C): 550.

IDA Survey: Scan type: TOFMS; Polarity: Negative or positive; Spray voltage (V): − 4500 or 5500; TOF start mass (Da): 60; TOF stop mass (Da): 1200; Accumulation time (s): 0.2; Declustering potential (V): − 60 or 60; Declustering potential spread (V): 0; Collision energy (V): − 10 or 10; Collision energy spread (V): 0.

IDA Dependent: Scan type: TOFMSMS; Polarity: Negative or positive; Spray voltage (V): − 4500 or 5500; Fragmentation mode: CID; TOF start mass (Da): 30; TOF stop mass (Da): 1250; Accumulation time (s): 0.02; Declustering potential (V): − 60 or 60; Declustering potential spread (V): 0; Collision energy (V): − 35 or 35; Collision energy spread (V): 15.

Differences in liver metabolite content between fructose exposing group and control group were described by fold regulation, P value generated from paired t-tests, and corrected p value generated from Benjamin Hochberg correction.

### Rats liver acetylation proteomics analysis

#### Protein extraction and peptide enzymatic hydrolysis

Powdered liver tissues were extracted using UA (8 M Urea, 100 mM Tris/HCl, pH 8.5) lysis method to extract proteins^16^, and then protein quantification was performed using the Bradford method. 20 µg of protein from each sample was added to 5 × loading buffer solution, and the boiling water bath was used for 5 min. 12.5% SDS-PAGE electrophoresis (constant flow 14 mA, 90 min) and Coomassie brilliant blue staining were performed. Added DTT to all samples to make the final concentration to 10 mM, placed them in a constant temperature mixer (600 rpm, 37 ℃) for 1.5 h, and cooled them to a room temperature. Later added IAA to the samples to make the final concentration to 50 mM and kept them in dark for 30 min. Later added 4 times the volume of 50 mM Tris HCl (pH 8.0), diluted the UA concentration to 2 M, and added trypsin in a 50:1 ratio of protein: trypsin by mass to digest proteins at 37 ℃ overnight (15–18 h). Added TFA to make the final concentration to 0.1%, and adjusted pH (≤ 3) by 10% TFA. Finally, applied C18SPECarrridge column to desalinate and freeze-dry peptide segments.

#### Acetylated peptide enrichment

Freeze-dried peptide samples were redissolved by 1.4 mL of precooled IAP buffer, and then were added pretreated Anti-Ac-K antibody beans (PTMScan Acetyl-Lysine Motif (Ac-K) Kit, Cell Signal Technology). Later incubated at 4 ℃ for 1.5 h, centrifuged at 3000 rpm for 30 s, and the supernatant were discarded. Anti-Ac-K antibody beans were cleaned 3 times with 1 mL precooled IAP buffer and then were cleaned 3 times with 1 mL precooled water again. Added 40 μL 0.15% TFA twice to cleaned anti-Ac-K antibody beans at room temperature for 10 min. And then centrifuged at 3000 rpm for 30 s, and the supernatant was desalinated with C18 STAGE Tips.

#### LC–MS/MS data collection

Each sample was separated using a nanoElute HPLC system with a nanoliter flow rate. Buffer solution A was a 0.1% formic acid aqueous solution, while solution B was a 0.1% formic acid acetonitrile aqueous solution. The chromatographic column was equilibrated with solution A, and the sample was separated through the column (homemade column, 25 cm ID 75 μm, 1.9 μm, C18) at a flow rate of 300 nL/min and the column temperature was 50 ℃.

After chromatographic separation, the sample was subjected to the timsTOF Pro mass spectrometer. The detection method was positive mode, and the ionization source voltage was 1.5 kV, both MS and MS/MS used TOF for detection and analysis. The mass spectrometry scanning range was set to 100–1700 m/z. The data collection adopted the Flat Accumulated Serial Fragmentation (PASEF) mode, and after the first level mass spectrometry collection, 10 times of PASEF mode collection was performed, with a cycle window time of 1.17 s. The dynamic exclusion time of the tandem mass spectrometry scan was set to 24 s to avoid repeated scanning of parent ion of MS/MS spectra of the charge from 0 to 5.

#### Protein identification and quantitative analysis

The original data of mass spectrometry was raw file, and MaxQuant software (version 1.5.3.17)^17^ was used for database checking, identification and quantitative analysis. Significantly modified peptide segments were screened and the up-regulated and down-regulated modified peptide segments were obtained on the criteria of fold regulation > 2 (up regulation greater than 2.0-fold or down regulation less than 0.5-fold) and Benjamin Hochberg corrected P value < 0.05. Then the GO and KEGG Pathway enrichment analysis were carried out according to the methods introduced in the references^18,19^.

### Correlation coefficients analysis among the differential genes, metabolites, and acetylated proteins in rat liver^15^

The correlation coefficients among the differential genes, metabolites, and acetylated proteins in rat liver were measured by Pearson correlation coefficient in R software (version: 4.3.1) and were visualized by heatmap in Heml software (Version: 1.0.3.7), which is a software dedicated to drawing heatmaps (https://hemi.biocuckoo.cn/).

### Ethics, consent and permissions

All subjects gave their informed written consent and the study was done in accordance with the Declaration of Helsinki. The study was approved by the Ethics Committees of the Affiliated Nanhua Hospital, University of South China (Ethical approval number: 2022-KY-167).

## Results

### Association between fructose exposure and amino acid metabolism in Chinese community residents

We screened total 1895 fasting serum samples from a community in southern Hunan province. Basic demography characteristics of the total population was shown in Table 1. Fructose can be detected in 1040 fasting serum samples (the detection rate of fructose was 54.88%) (Table 2). In the residents who can detect fructose, the descriptive statistical indicators such as the mean, the median, the highest and lowest of serum fasting fructose content were 116.96 μg/mL, 16.41 μg/mL, 1704.64 μg/mL and 0.11 μg/mL, respectively (Table 2). These data indicated individual exposure levels of fructose varied obviously.

**Table 2:** Fasting serum fructose exposure level of 1895 community residents.

	Not detected	Detected
Sample size (n)	855	1040
Q1 (μg/mL)	/	7.64
Q2 (μg/mL)	/	16.41
Q3 (μg/mL)	/	41.21
The highest level of serum fasting fructose (μg/mL)	/	1704.64
The lowest level of serum fasting fructose (μg/mL)	/	0.11
Average level of serum fasting fructose (μg/mL)	/	116.96

We further screened 260 high fructose exposed population from these 1040 subjects by quartile method according to their fructose exposure level in serum, and 260 controls were strictly matched based on age and gender from the 855 subjects who had not been detected for fructose. The descriptive statistical indicators of the residents whose fasting serum fructose exposure level were in the larger quartile were 426.79 μg/mL, 191.95 μg/mL, 1704.64 μg/mL and 41.29 μg/mL, respectively (Table 3).

**Table 3:** Amino acid metabolic difference between high fructose exposure population in the larger quartile and the accurately matched control.

	Control	High fructose concentration group	P value	Corrected P value^#^
Mean/median of fructose (μg/mL)	0	426.79/191.95		
The highest/lowest level of fructose (μg/mL)	0	1704.64/41.29		
Age	68.65 ± 10.00^a^	68.66 ± 10.03^a^	0.99	
Gender (Male/Female)	104/156	104/156		
Diabetes (n)	45	58	0.15	
Uric acid (μmol/L)	324.30 ± 73.28^a^	324.91 ± 78.08^a^	0.93	
Height (cm)	158.46 ± 8.79^a^	158.04 ± 7.80^a^	0.57	
Weight (Kg)	59.52 ± 10.62^a^	58.59 ± 10.46^a^	0.32	
Waist circumference (cm)	82.23 ± 9.85^a^	80.91 ± 10.42^a^	0.14	
BMI (kg/cm^2^)	23.63 ± 3.29^a^	23.36 ± 3.16^a^	0.34	
Left systolic blood pressure (mm/Hg)	138.5 ± 18.63^a^	139.54 ± 18.66^a^	0.53	
Left diastolic blood pressure (mm/Hg)	74.38 ± 7.89^a^	74.42 ± 7.76^a^	0.95	
Right systolic blood pressure (mm/Hg)	141.88 ± 18.54^a^	142.94 ± 18.33^a^	0.51	
Right diastolic blood pressure (mm/Hg)	77.72 ± 7.67^a^	77.99 ± 7.67^a^	0.69	
Hemoglobin (g/L)	131.79 ± 15.02^a^	132.10 ± 14.26^a^	0.81	
White blood cells (× 10^9^/L)	6.87 ± 4.21^a^	6.62 ± 1.74^a^	0.38	
Platelet (× 10^9^/L)	217.39 ± 121.57^a^	208.75 ± 61.64^a^	0.31	
Fasting blood glucose (mmol/L)	6.30 ± 1.61^a^	6.93 ± 2.68^a^	0.10*10^–2^	0.72*10^–2^
Serum ALT (U/L)	22.92 ± 17.37^a^	22.42 ± 12.74^a^	0.71	
Serum AST (U/L)	23.55 ± 12.34^a^	22.66 ± 9.99^a^	0.37	
Total_Bilirubin (μmol/L)	14.01 ± 4.72^a^	13.52 ± 4.65^a^	0.23	
Serum creatinine (μmol/L)	79.37 ± 31.37^a^	73.56 ± 21.16^a^	0.01	0.05
Blood urea nitrogen (mmol/L)	5.77 ± 1.80^a^	5.61 ± 1.52^a^	0.28	
Total cholesterol (mmol/L)	4.62 ± 0.95^a^	4.76 ± 0.99^a^	0.09	
Triglycerides (mmol/L)	1.48 ± 1.05^a^	1.82 ± 1.60^a^	0.4*10^–2^	0.02
Serum low density lipoprotein cholesterol (mmol/L)	2.60 ± 0.64^a^	2.60 ± 0.68^a^	0.97	
Serum high density lipoprotein cholesterol (mmol/L)	1.49 ± 0.31^a^	1.45 ± 0.32^a^	0.17	
Heart rate	72.14 ± 10.05^a^	74.67 ± 11.88^a^	0.01	0.04
His (μg/mL)	15.53 ± 4.92^a^	15.74 ± 5.12^a^	0.64	
Hyp (μg/mL)	3.50 ± 2.56^a^	3.04 ± 2.12^a^	0.03	0.09
3MHis (μg/mL)	0.45 ± 0.96^a^	0.32 ± 0.44^a^	0.05	0.15
1MHis (μg/mL)	1.05 ± 0.80^a^	0.74 ± 0.40^a^	6.74*10^–8^	2.33*10^–6^
PEtN (μg/mL)	0.30 ± 0.21^a^	0.22 ± 0.16^a^	4.25*10^–7^	9.78*10^–6^
Asn (μg/mL)	7.09 ± 1.85^a^	7.23 ± 2.50^a^	0.45	
Arg (μg/mL)	23.52 ± 24.19^a^	16.89 ± 10.85^a^	7.00*10^–5^	0.80*10^–4^
Car (μg/mL)	0.07 ± 0.05^a^	0.08 ± 0.06^a^	0.07	
Tau (μg/mL)	21.34 ± 6.78^a^	21.30 ± 8.16^a^	0.95	
Ans (μg/mL)	0.19 ± 0.15^a^	0.23 ± 0.16^a^	0.88*10^–2^	0.04
Ser (μg/mL)	17.15 ± 4.12^a^	17.14 ± 5.67^a^	0.98	
Gln (μg/mL)	82.94 ± 20.21^a^	77.06 ± 21.17^a^	0.13*10^–2^	0.01
Asa (μg/mL)	0.08 ± 0.12^a^	0.10 ± 0.06^a^	0.11*10^–2^	0.01
Gly (μg/mL)	24.25 ± 8.24^a^	24.86 ± 9.57^a^	0.43	
EtN (μg/mL)	0.66 ± 0.27^a^	0.75 ± 0.33^a^	0.98*10^–3^	0.01
Asp (μg/mL)	5.05 ± 1.78^a^	5.68 ± 2.34^a^	0.67*10^–3^	0.01
Cit (μg/mL)	7.32 ± 2.86^a^	6.93 ± 2.66^a^	0.11	
Sar (μg/mL)	0.15 ± 0.07^a^	0.13 ± 0.06^a^	0.03	0.10
Glu (μg/mL)	18.48 ± 8.57^a^	21.79 ± 11.39^a^	0.21*10^–3^	0.2*10^–2^
bAla (μg/mL)	0.34 ± 0.14^a^	0.32 ± 0.13^a^	0.12	
Thr (μg/mL)	16.11 ± 4.43^a^	15.53 ± 5.04^a^	0.16	
Ala (μg/mL)	43.21 ± 12.73^a^	43.44 ± 14.89^a^	0.85	
Hcit (μg/mL)	0.08 ± 0.06^a^	0.08 ± 0.05^a^	0.65	
GABA (μg/mL)	0.03 ± 0.02^a^	0.02 ± 0.01^a^	2.86*10^–6^	4.93*10^–5^
Aad (μg/mL)	0.17 ± 0.07^a^	0.15 ± 0.06^a^	0.13*10^–2^	0.73*10^–2^
Hyl (μg/mL)	0.11 ± 0.10^a^	0.05 ± 0.05^a^	4.56*10^–14^	3.14*10^–12^
bAib (μg/mL)	0.27 ± 0.26^a^	0.28 ± 0.28^a^	0.91	
Pro (μg/mL)	21.51 ± 7.72^a^	21.57 ± 8.20^a^	0.93	
Cth (μg/mL)	0.13 ± 0.16^a^	0.10 ± 0.15^a^	0.07	
Abu (μg/mL)	1.88 ± 0.67^a^	1.85 ± 0.78^a^	0.64	
Cys (μg/mL)	14.71 ± 5.72^a^	12.82 ± 4.35^a^	2.80*10^–5^	0.39*10^–3^
Tyr (μg/mL)	13.39 ± 3.73^a^	12.64 ± 3.88^a^	0.03	0.09
Met (μg/mL)	3.85 ± 1.02^a^	3.87 ± 1.27^a^	0.87	
Val (μg/mL)	29.23 ± 7.45^a^	28.62 ± 8.34^a^	0.38	
Ile (μg/mL)	9.46 ± 2.58^a^	9.07 ± 2.92^a^	0.11	
Leu (μg/mL)	19.47 ± 4.81^a^	19.64 ± 7.43^a^	0.75	
Phe (μg/mL)	13.49 ± 3.49^a^	14.00 ± 4.36^a^	0.14	
Trp (μg/mL)	11.26 ± 2.87^a^	10.90 ± 3.44^a^	0.19	
Lys (μg/mL)	29.07 ± 6.97^a^	29.06 ± 7.93^a^	0.98	

Abbr.: Histidine (His); 1-Methyl-l-histidine (1MHis); 3-Methyl- l-histidine (3MHis); Hydroxy- proline (Hyp); Asparagine (Asn); Phosphorylethanolamine (PEtN); Arginine (Arg); Carnosine (Car); Anserine (Ans); Argininosuccinic acid (Asa); Serine (Ser); Taurine (Tau); Glutamine (Gln); Ethanolamine (EtN); Glycine (Gly); Sarcosine (Sar); beta-Alanine (bAla); Threonine (Thr); Aspartic acid (Asp); Glutamic acid (Glu); Citrulline (Cit); Alanine (Ala); gamma-Aminobutyric acid (GABA); Aminoisobutyric acid (bAib); Proline (Pro); Aminoadipic acid (aAd); 5-Hydroxylysine (Hyl); Homocitrulline (Hcit); 2-Aminobutyric acid (Abu); Valine (Val); Methionine (Met); Tyrosine (Tyr); Cystathionine (Cth); Cystine (Cys); Leucine (Leu); Isoleucine (Ile); Phenylalanine (Phe); Tryptophan (Trp); Lysine (Lys).

^a^Values are presented as mean ± standard deviation.

^#^P value with Benjamin Hochberg correction.

We conducted amino acid targeted metabolomics testing on these 1895 subjects using UPLC-QqQMS. In high fructose exposing group, fasting blood glucose, triglycerides, heart rate, and amino acids of arginosuccinate (Asa), ethanolamine (EtN), aspartic acid (Asp), and glutamic acid (Glu) elevated significantly, while serum creatinine and amino acids of 1-methyl-l-histidine (1MHis), phosphorylethanolamine (PEtN), arginine (Arg), glutamine (Gln), gamma-aminobutyric acid (GABA), amino- adipic acid (aAd), 5-hydroxylysine (Hyl) and cystine (Cys) decreased significantly (Table 3). Amino acid metabolism was further tested with regard to gender differences (Supplementary table 3 and 4). Fructose exposure significantly increased the fasting blood glucose, heart rate, and Car, but decreased 1MHis, Arg, GABA, Hyl, and Cys in the male residents (Supplementary table 3). In the female residents, fructose exposure significantly increased Ans, Asa, EtN, Asp and Glu, but decreased 1MHis, PEtN, Gln, Aad and Hyl (Supplementary table 4). These findings suggested that the health damage caused by fructose may be related to gender.

### Effects on metabolome of liver extract and liver genes of amino acid metabolic enzymes after fructose exposure in rat

The high-resolution mass spectrometry based liver nontargeted metabolomics shown that fructose exposure caused significant increase (fold regulation > 2, and corrected P value ≤ 0.05) in 5 metabolites such as calcidiol, 19,20-DiHDPA, PGF2aethanolamide, stearoylethanolamide, and palmitoylethanolamide and significant decrease (fold regulation < 0.5, and corrected P value ≤ 0.05) in 5 metabolites such as 5-hydroxyindoleacetaldehyde, indoleacetic acid, guanidino- succinic acid, glyceraldehyde and taurodeoxycholic acid 3-glucuronide (Table 4).

**Table 4:** Metabolites identified by high-resolution mass spectrometry with significant changes caused by fructose exposure in liver.

Component name	Formula	Retention time	Adduct/charge	Precursor mass	Fold regulation	P value	Corrected P value^#^
Calcidiol	C27H44O2	15.27	[M + H]^+^	401.341	4.45	0.27*10^–2^	0.04
19,20-DiHDPA	C22H34O4	9.67	[M + H]^+^	363.253	4.26	0.51*10^–2^	0.05
PGF2a ethanolamide	C22H39NO5	9.69	[M + H]^+^	398.29	3.56	0.73*10^–2^	0.05
Hydroxyindole-acetaldehyde	C10H9NO2	1.77	[M + H]^+^	176.071	0.47	0.82*10^–2^	0.05
Indoleacetic acid	C10H9NO2	1.77	[M + H]^+^	176.071	0.47	0.81*10^–2^	0.05
Stearoyl-ethanolamide	C20H41NO2	14.38	[M + H]^+^	328.321	2.83	0.87*10^–2^	0.05
Guanidino-succinic acid	C5H9N3O4	1.76	[M + H]^+^	176.067	0.48	0.01	0.05
Glyceraldehyde	C3H6O3	2.22	[M-H]^-^	89.024	0.48	0.01	0.04
Palmitoyl-ethanolamide	C18H37NO2	13.57	[M + H]^+^	300.29	2.02	0.01	0.04
Taurodeoxycholic acid 3-glucuronide	C32H53NO12S	9.37	[M-H]^-^	674.322	0.02	0.02	0.05

^#^P value with Benjamin Hochberg correction.

We tested 84 genes of amino acid metabolic enzymes in the liver after 6 months 5% fructose exposure using RT^2^ Profiler PCR Arrays to further explore the mechanism of fructose interfering with amino acid metabolism in the body. The list of 84 amino acid metabolism genes were shown in supplementary table 2. Twenty-one amino acid metabolic genes such as ***Aadat***, ***Aasdhppt***, ***Abat***, ***Acaa2***, ***Agxt*, *Aldh2***, ***Aldh6a1***, ***Aspa***, ***Bckdhb*, *Cndp1***, ***Dao***, ***Dbt*, *Ddc***, ***Dmgdh****, ****Gldc****, ****Gnmt*, *Hadh*, *Hadhb*, *Mcee****, ****Pah**** and ****Plod3*** changed significantly after fructose exposure (Table 5). After Benjamin Hochberg correction, fructose still caused significant changes in 4 amino acid metabolic genes (***Aspa*, *Cndp1*, *Dbt*, *Dmgdh***) (Table 5). ***Cndp1*** and ***Dbt*** significantly increased while ***Aspa*** and ***Dmgdh*** significantly decreased.

**Table 5:** Significant change in genes expression of amino acid metabolism related enzymes after exposure to fructose in rat liver.

Gene Symbol	Fold Change	P Value	Corrected P value
*Aadat*	0.55	0.03	0.17
*Aasdhppt*	0.79	0.04	0.17
*Aass*	1.88	0.12	0.26
*Abat*	0.79	0.05	0.18
*Acaa2*	1.88	0.04	0.18
*Acadm*	1.10	0.49	0.64
*Acads*	0.99	0.86	0.91
*Acadsb*	1.10	0.46	0.61
*Acat2*	0.61	0.20	0.32
*Adh6a*	1.12	0.53	0.67
*Adsl*	1.40	0.14	0.28
*Adss*	1.19	0.09	0.26
*Agxt*	0.43	0.03	0.20
*Alas1*	1.12	0.59	0.68
*Aldh2*	1.12	0.04	0.17
*Aldh3b1*	1.25	0.52	0.67
*Aldh5a1*	0.97	0.70	0.79
*Aldh6a1*	1.57	0.04	0.18
*Amt*	1.32	0.10	0.26
*Aoc1*	1.00	0.88	0.91
*Aoc3*	2.44	0.39	0.54
*Ash1l*	0.99	0.88	0.91
*Asns*	1.24	0.43	0.59
*Aspa*	0.45	0.29*10^–2^	0.05
*Bbox1*	0.78	0.09	0.26
*Bcat1*	2.08	0.27	0.42
*Bckdhb*	0.41	**0.03**	0.18
*Bhmt*	0.44	0.13	0.28
*Chdh*	0.80	0.11	0.25
*Cndp1*	1.61	0.10*10^–2^	0.02
*Comt*	0.57	0.14	0.27
*Dao*	1.64	0.01	0.14
*Dbh*	3.12	0.12	0.26
*Dbt*	1.34	0.58*10^–3^	0.02
*Ddc*	0.62	0.03	0.18
*Dld*	0.94	0.59	0.69
*Dlst*	1.25	0.09	0.26
*Dmgdh*	0.68	0.8*10^–3^	0.02
*Echs1*	1.03	0.77	0.84
*Fah*	1.31	0.10	0.26
*Ftcd*	1.30	0.16	0.30
*Gad2*	3.46	0.21	0.34
*Gcat*	0.82	0.11	0.26
*Gcdh*	1.50	0.11	0.26
*Gldc*	0.42	0.01	0.16
*Gnmt*	0.57	0.04	0.19
*Got1*	1.43	0.18	0.31
*Gpt*	1.24	0.31	0.46
*Hadh*	1.43	0.03	0.18
*Hadhb*	1.38	0.03	0.16
*Hdc*	0.63	0.36	0.52
*Hgd*	1.59	0.15	0.28
*Hibadh*	0.50	0.08	0.25
*Hibch*	1.43	0.17	0.30
*Hnmt*	1.44	0.57	0.70
*Hpd*	1.17	0.19	0.32
*Hsd17b10*	0.79	0.18	0.31
*Iars*	1.35	0.06	0.20
*Lcmt2*	0.50	0.08	0.26
*Maoa*	1.01	0.99	0.97
*Mcee*	1.41	0.02	0.19
*Mif*	1.22	0.40	0.56
*Mmut*	1.23	0.09	0.26
*Ogdhl*	0.68	0.58	0.69
*Pah*	1.59	0.01	0.13
*Pcca*	1.18	0.07	0.25
*Pdha2*	0.61	0.30	0.45
*Phgdh*	1.26	0.57	0.69
*Pipox*	0.88	0.17	0.30
*Plod3*	1.48	0.02	0.19
*Prdx6*	0.90	0.51	0.67
*Psat1*	0.92	0.79	0.85
*Psph*	0.70	0.10	0.26
*Sardh*	0.99	0.95	0.95
*Sds*	0.63	0.13	0.27
*Shmt2*	1.04	0.60	0.68
*Srr*	0.90	0.57	0.69
*Th*	2.08	0.89	0.90
*Tmlhe*	1.20	0.30	0.46
*Tpo*	7.02	0.39	0.55
*Tyr*	1.87	0.73	0.80
*Tyrp1*	1.55	0.86	0.90
*Vars2*	1.47	0.15	0.27
*Wbscr22*	2.37	0.06	0.22

Abbr.:Aminoadipate aminotransferase (Aadat); Aminoadipate-semialdehyde dehydrogenase- phosphopantetheinyl transferase (Aasdhppt); Aminoadipate-semialdehyde synthase (Aass); 4- aminobutyrate aminotransferase (Abat); Amiloride binding protein 1 (amine oxidase, copper- containing) (Aoc1); Acetyl-Coenzyme A acyltransferase 2 (Acaa2); Acyl-Coenzyme A dehydro- genase, C-4 to C-12 straight chain (Acadm); Acyl-Coenzyme A dehydrogenase, C-2 to C-3 short chain (Acads); Acyl-Coenzyme A dehydrogenase, short/branched chain (Acadsb); Acetyl- Coenzyme A acetyltransferase 3 (Acat2); Alcohol dehydrogenase 6A (class V) (Adh6a); Adenylosuccinate lyase (Adsl); Adenylosuccinate synthase (Adss); Alanine-glyoxylate aminotransferase (Agxt); Aminolevulinate, delta-, synthase 1 (Alas1); Aldehyde dehydrogenase 2 family (mitochondrial) (Aldh2); Aldehyde dehydrogenase 3 family, member B1 (Aldh3b1); Aldehyde dehydrogenase 5 family, member A1 (Aldh5a1); Aldehyde dehydrogenase 6 family, member A1 (Aldh6a1); Aminomethyltransferase (Amt); Amine oxidase, copper containing 3 (vascular adhesion protein 1) (Aoc3); Ash1 (absent, small, or homeotic)-like (Drosophila) (Ash1l); Asparagine synthetase (Asns); Aspartoacylase (Aspa); Butyrobetaine (gamma), 2-oxoglutarate dioxygenase (gamma-butyrobetaine hydroxylase) 1 (Bbox1); Branched chain aminotransferase 1, cytosolic (Bcat1); Branched chain keto acid dehydrogenase E1, beta polypeptide (Bckdhb); Betaine-homocysteine methyltransferase (Bhmt); Choline dehydrogenase (Chdh); Carnosine dipeptidase 1 (metallopeptidase M20 family) (Cndp1); Catechol-*O*-methyltransferase (Comt); D-amino-acid oxidase (Dao); Dopamine beta-hydroxylase (dopamine beta-monooxygenase) (Dbh); Dihydrolipoamide branched chain transacylase E2 (Dbt); Dopa decarboxylase (aromatic L-amino acid decarboxylase) (Ddc); Dihydrolipoamide dehydrogenase (Dld); Dihydrolipoamide S-succinyltransferase (E2 component of 2-oxo-glutarate complex) (Dlst); Dimethylglycine dehydrogenase (Dmgdh); Enoyl Coenzyme A hydratase, short chain, 1, mitochondrial (Echs1); Fumarylacetoacetate hydrolase (Fah); Formiminotransferase cyclodeaminase (Ftcd); Glutamate decarboxylase 2 (Gad2); Glycine C-acetyltransferase (2-amino-3-ketobutyrate-coenzyme A ligase) (Gcat); Glutaryl-Coenzyme A dehydrogenase (Gcdh); Glycine dehydrogenase (decarboxylating) (Gldc); Glycine N-methyltransferase (Gnmt); Glutamic-oxaloacetic transaminase 1, soluble (aspartate aminotransferase 1) (Got1); Glutamic-pyruvate transaminase (alanine aminotransferase) (Gpt); Hydroxyacyl-Coenzyme A dehydrogenase (Hadh); Hydroxyacyl-Coenzyme A dehydro- genase/3-ketoacyl-Coenzyme A thiolase/enoyl-Coenzyme A hydratase (trifunctional protein), beta subunit (Hadhb); Histidine decarboxylase (Hdc); Homogentisate 1, 2-dioxygenase (Hgd); 3-hydroxyisobutyrate dehydrogenase (Hibadh); 3-hydroxyisobutyryl-Coenzyme A hydrolase (Hibch); Histamine *N*-methyltransferase (Hnmt); 4-hydroxyphenylpyruvate dioxygenase (Hpd); Hydroxysteroid (17-beta) dehydrogenase 10 (Hsd17b10); Isoleucyl-tRNA synthetase (Iars); Leucine carboxyl methyltransferase 2 (Lcmt2); Monoamine oxidase A (Maoa); Methylmalonyl CoA epimerase (Mcee); Macrophage migration inhibitory factor (Mif); Methylmalonyl-CoA mutase (Mmut); Oxoglutarate dehydrogenase-like (Ogdhl); Phenylalanine hydroxylase (Pah); Propionyl-coenzyme A carboxylase, alpha polypeptide (Pcca); Pyruvate dehydrogenase (lipoamide) alpha 2 (Pdha2); Phosphoglycerate dehydrogenase (Phgdh); Pipecolic acid oxidase (Pipox); Procollagen-lysine, 2-oxoglutarate 5-dioxygenase 3 (Plod3); Peroxiredoxin 6 (Prdx6); Phosphoserine aminotransferase 1 (Psat1); Phosphoserine phosphatase (Psph); Sarcosine de- hydrogenase (Sardh); Serine dehydratase (Sds); Serine hydroxymethyltransferase 2 (mito- chondrial) (Shmt2); Serine racemase (***Srr***); Tyrosine hydroxylase (Th); Trimethyllysine hydroxylase, epsilon (Tmlhe); Thyroid peroxidase (Tpo); Tyrosinase (Tyr); Tyrosinase-related protein 1 (Tyrp1); Valyl-tRNA synthetase 2, mitochondrial (putative) (Vars2); Williams Beuren syndrome chromosome region 22 (Wbscr22).

### Effects on liver acetylated proteome after exposure to fructose in rat

**Figure 1:**
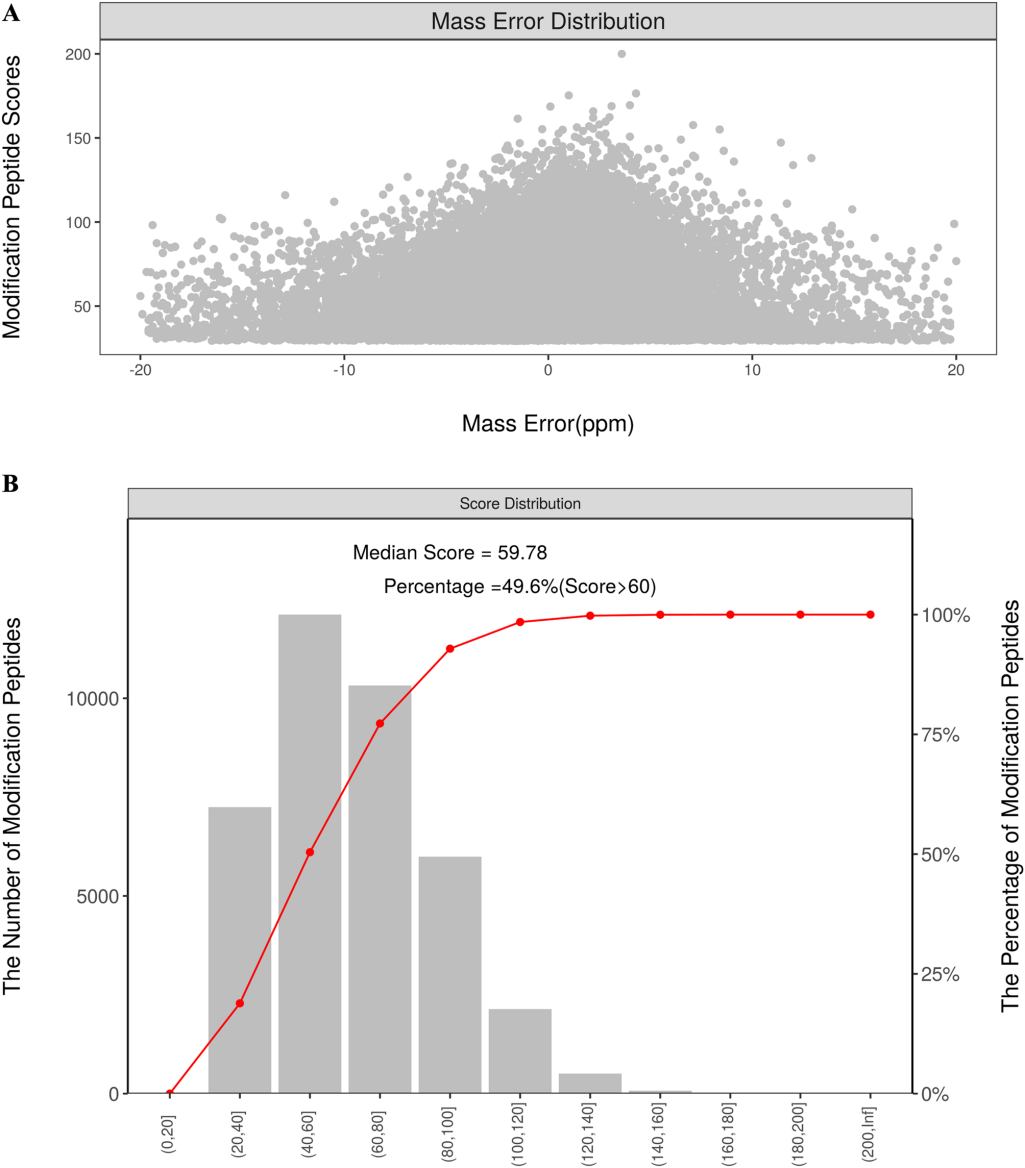
Quality control for liver acetylation proteomics analysis. (**A**) Distribution map of ion mass deviation for modified peptide segments; (**B**) distribution map of modified peptide ion scores.

**Figure 2:**
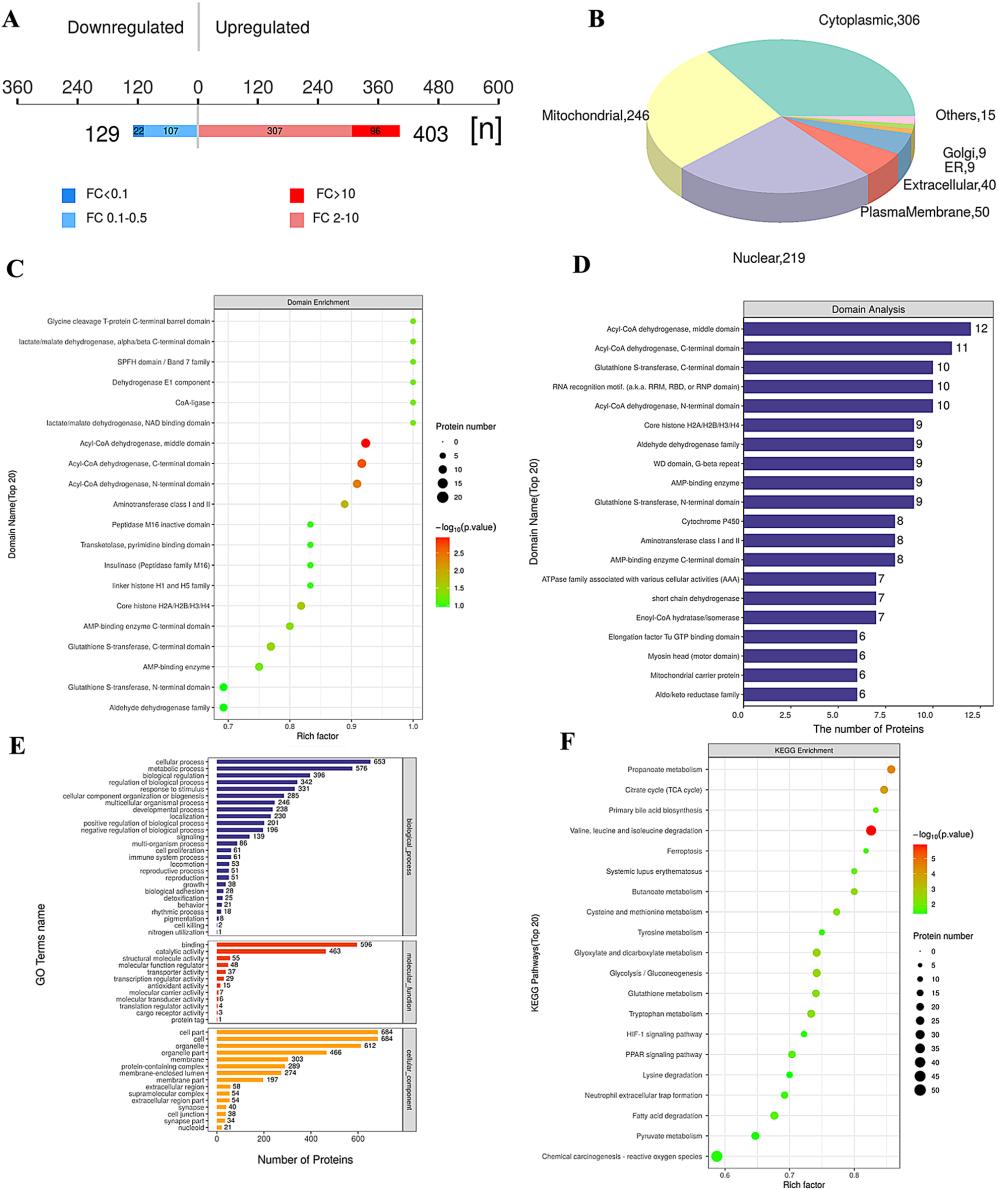
Bioinformatics analysis for liver acetylation proteomics. (**A**) Histogram of quantitative differences in acetylated peptide segments (the fructose exposure group vs the control group); (**B**) subcellular localization pie chart of proteins that differentially expressed modified peptide segments belonging to; (**C**) structural domain enrichment analysis diagram; (**D**) analysis of protein domains that differentially expressed modified peptide segments belonging to; (**E**) GO annotation of proteins that differentially expressed acetylated peptide segments corresponding to (level 2); (**F**) KEGG pathway enrichment map of proteins that differentially expressed acetylated peptide corresponding to.

Using acetylated 4D Label free quantitative proteomics technology, we further evaluated the impact of fructose exposure on liver acetylated proteome. As shown in Fig. 1A, the mass deviation of all identified acetylated peptide segments is mainly distributed within 10 ppm, indicating that the identification results are accurate and reliable. Then, using the rigorous analysis tool Andromeda, the MS profile data was analyzed to obtain the score for each MS_2_. Figure 1B showed that the Andromeda score of MS_2_ is relatively ideal, with over 49.6% peptides above 20 points, and the median peptide score is 59.78 points. These results indicated that our mass spectrometry data is relatively reliable. Figure 2 displayed bioinformatics analysis for liver acetylation proteomics analysis. A total of 403 peptide segments were upregulated and 129 peptide segments were downregulated (Fig. 2A). Among them, there were 306 proteins in the cytoplasm, 246 proteins in mitochondria, and 219 proteins in the nucleus with acetylation modifications (Fig. 2B). The protein domains with the most significant acetylated modified level were middle domain, C-terminal domain and N-terminal domain of acyl-CoA dehydrogenase (Fig. 2C, D). GO annotation analysis displayed biological processes of cellular process, metabolic process and biological regulation, molecular function of binding, catalytic activity and structural molecule activity, and cellular components of cell part, cell and organelle changed significantly (Fig. 2E). And KEGG pathway enrichment map displayed that valine, leucine and isoleucine degradation changed most and followed by propanoate metabolism and citrate cycle (TCA cycle) (Fig. 2F). These bioinfor- matics results provided valuable clues for further research in the mechanism of fructose interference in amino acid metabolism.

In particular, we found a significant increase in acetylation modifications of 46 specific proteins (Table 6). Among them, proteins involved in lipid, energy, and amino acid metabolism such as peroxisomal trans-2-enoyl-CoA reductase (**Pecr**), trans- ketolase (**Tkt**), ATP synthase F1 subunit alpha (**Atp5f1a**), solute carrier family 25 member 12 (**Slc25a12**), 3-hydroxy isobutyryl-CoA hydrolase (**Hibch**), acetyl-CoA acetyl-transferase 1 (**Acat1**), acyl-CoA dehydrogenase, short/branched chain (**Acadsb)**, glutathione S-transferase, theta 2 (**Gstt2)**, acetyl-coenzyme a acyltrans- ferase 2 (**Acaa2)**, as well as isovaleryl-coa dehydrogenase (**Ivd)** had the highest level of acetylation after fructose exposure.

**Table 6:** Significant change in protein acetylated modification after fructose exposure in rat liver.

Protein name	Acetylated location	MotifSequence	Fold regulation	P value	Corrected P value^#^
Pecr	K92	EVNNLVKSTLAKY	135.37	0.5*10^–3^	0.04
Tkt	K236	QVDVYQKRCEAFG	91.06	0.6*10^–3^	0.04
Atp5f1a	K531	NIRSDGKISEQSD	88.89	0.1*10^–3^	0.02
Slc25a12	K384	APEKAIKLTVNDF	85.48	0.8*10^–4^	0.01
Hibch	K352	RAVLIDKDQTPKW	78.53	0.3*10^–7^	0.3*10^–2^
Acat1	K199	CAENTAKKLSISR	67.83	0.1*10^–4^	0.7*10^–2^
Acadsb	K284	KIGHGYKYAIGSL	50.29	0.8*10^–4^	0.01
Gstt2	K52	SQVNCLKKVPVLK	49.30	0.2*10^–4^	0.8*10^–2^
Acaa2	K269	SEDAVKKHNFTPL	33.25	0.7*10^–4^	0.01
Ivd	K240	GFSTSKKLDKLGM	32.87	0.1*10^–4^	0.5*10^–2^
Got2	K302	KDAEEAKRVESQL	31.34	0.3*10^–4^	0.8*10^–2^
Hsd17b10	K52	EGETEAKKLGGNC	28.14	0.5*10^–3^	0.04
Glul	K333	RIVGQEKKGYFED	26.81	0.1*10^–4^	0.5*10^–2^
Dld	K267	FQRILQKQGFKFK	23.13	0.7*10^–4^	0.01
Suox	K495	WRIWQLKAHVPAE	21.67	0.4*10^–3^	0.04
Tcp1	K400	DALCVVKRVLESK	19.22	0.1*10^–4^	0.6*10^–2^
Myh4	K914	RCDQLIKTKIQLE	18.21	0.9*10^–4^	0.01
Suclg2	K278	IFAMDDKSENEPI	17.09	0.1*10^–3^	0.02
Mybpc1	K479	GKEVCLKCEISEN	16.31	0.2*10^–3^	0.03
Pdhx	K488	RFRPVLKLTEDEE	15.70	0.2*10^–7^	0.5*10^–2^
Farsb	K317	ADLINKKVGIKET	14.61	0.3*10^–3^	0.03
Hadh	K249	DIDTAMKLGAGYP	13.98	0.3*10^–3^	0.03
Plec	K768	QDAQDEKEQLNEY	13.95	0.1*10^–4^	0.5*10^–2^
H1f2	K64	SLAALKKALAAAG	13.27	0.6*10^–3^	0.04
Letmd1	K209	ALVSNEKLRWHLE	12.49	0.3*10^–4^	0.9*10^–2^
Slc25a1	K268	CGVQILKNEGPKA	9.46	0.5*10^–3^	0.04
Hsd17b4	K275	VRDNWVKICDFSN	9.34	0.6*10^–4^	0.01
Hspd1	K359	DDAMLLKGKGDKA	9.23	0.8*10^–3^	0.05
Ak3	K165	IQREDDKPETVIK	8.56	0.4*10^–7^	0.3*10^–2^
Hadha	K411	FKGLNDKVKKKAL	8.53	0.6*10^–3^	0.04
Nln	K134	ETCDLEKIKPEAR	7.75	0.7*10^–3^	0.05
Snd1	K514	GDTQKAKQFLPFL	7.58	0.7*10^–3^	0.05
Idh2	K45	YAEKRIKVEKPVV	7.26	0.2*10^–3^	0.03
Bckdhb	K293	ASMAQEKLGVSCE	7.13	0.4*10^–2^	0.05
Macroh2a1	K234	LGSTLEKKGGKEF	7.07	0.6*10^–4^	0.01
Glrx5	K147	SALIDEKDQDSK_	6.93	0.7*10^–3^	0.05
Clpx	K475	HQDIEEKDRLLRH	6.64	0.2*10^–3^	0.03
Sardh	K885	VSLDFVKNGDYAL	6.53	0.1*10^–3^	0.02
Aldh1l2	K227	TYEGIQKKENAEI	5.47	0.1*10^–4^	0.7*10^–2^
Psmd11	K382	SLADFEKALTDYR	4.84	0.9*10^–3^	0.05
Cs	K321	LQKEVGKDVSDEK	4.69	0.2*10^–3^	0.03
Gmppa	K384	SLFKDGKLLPAIT	4.18	0.6*10^–3^	0.04
Ndufa10l1	K191	DHYNEIKRLTLPE	4.11	0.1*10^–3^	0.02
Pygl	K724	DVAALDKKGYEAK	3.45	0.4*10^–3^	0.04
Hspe1	K35	GIMLPEKSQGKVL	2.99	0.5*10^–3^	0.04
Bckdk	K233	PKKIIEKWVDFAR	2.01	0.5*10^–3^	0.04
Trip13	K243	QDLIDDKEALVFV	0.43	0.3*10^–3^	0.03
Otc	K231	PDPNIVKLAEQYA	0.21	0.3*10^–3^	0.03

Abbr.: peroxisomal trans-2-enoyl-CoA reductase (Pecr); transketolase (Tkt); ATP synthase F1 subunit alpha (Atp5f1a); solute carrier family 25 member 12 (Slc25a12); 3-hydroxyisobutyryl- CoA hydrolase (Hibch); acetyl-CoA acetyltransferase 1 (Acat1); acyl-CoA dehydrogenase, short/branched chain (Acadsb); glutathione S-transferase, theta 2 (Gstt2); acetyl-CoA acyl- transferase 2 (Acaa2); isovaleryl-CoA dehydrogenase (Ivd); glutamic-oxaloacetic transaminase 2 (Got2); hydroxysteroid (17-beta) dehydrogenase 10 (Hsd17b10); glutamate-ammonia ligase (Glul); dihydrolipoamide dehydrogenase (Dld); sulfite oxidase (Suox); t-complex 1 (Tcp1); myosin heavy chain 4 (Myh4); succinate-CoA ligase, GDP-forming, beta subunit (Suclg2); myosin binding protein C, slow type (Mybpc1); pyruvate dehydrogenase complex, component X (Pdhx); phenylalanyl-tRNA synthetase subunit beta (Farsb); hydroxyacyl-CoA dehydrogenase (Hadh); plectin (Plec); H1.2 linker histone, cluster member (H1f2); LETM1 domain containing 1 (Letmd1); solute carrier family 25 member 1 (Slc25a1); hydroxysteroid (17-beta) dehydrogenase 4 (Hsd17b4); heat shock protein family D (Hsp60) member 1 (Hspd1); adenylate kinase 3 (Ak3); hydroxyacyl-CoA dehydrogenase trifunctional multienzyme complex subunit alpha (Hadha); neurolysin (Nln); staphylococcal nuclease and tudor domain containing 1 (Snd1); isocitrate dehydrogenase (NADP( +)) 2 (Idh2); branched chain keto acid dehydrogenase E1 subunit beta (Bckdhb); macroH2A.1 histone (Macroh2a1); glutaredoxin 5 (Glrx5); caseinolytic mitochondrial matrix peptidase chaperone subunit (Clpx); sarcosine dehydrogenase (Sardh); aldehyde dehydrogenase 1 family, member L2 (Aldh1l2); proteasome 26S subunit, non-ATPase 11 (Psmd11); citrate synthase (Cs); GDP-mannose pyrophosphorylase A (Gmppa); NADH dehydrogenase (ubiquinone) 1 alpha subcomplex 10-like 1 (Ndufa10l1); glycogen phosphorylase L (Pygl); heat shock protein family E (Hsp10) member 1 (Hspe1); branched chain ketoacid dehydrogenase kinase (Bckdk); thyroid hormone receptor interactor 13 (Trip13); ornithine carbamoyltransferase (Otc).

^#^P value with Benjamin Hochberg correction.

### Correlation coefficients analysis among the differential genes, metabolites, and acetylated proteins in rat liver

**Figure 3:**
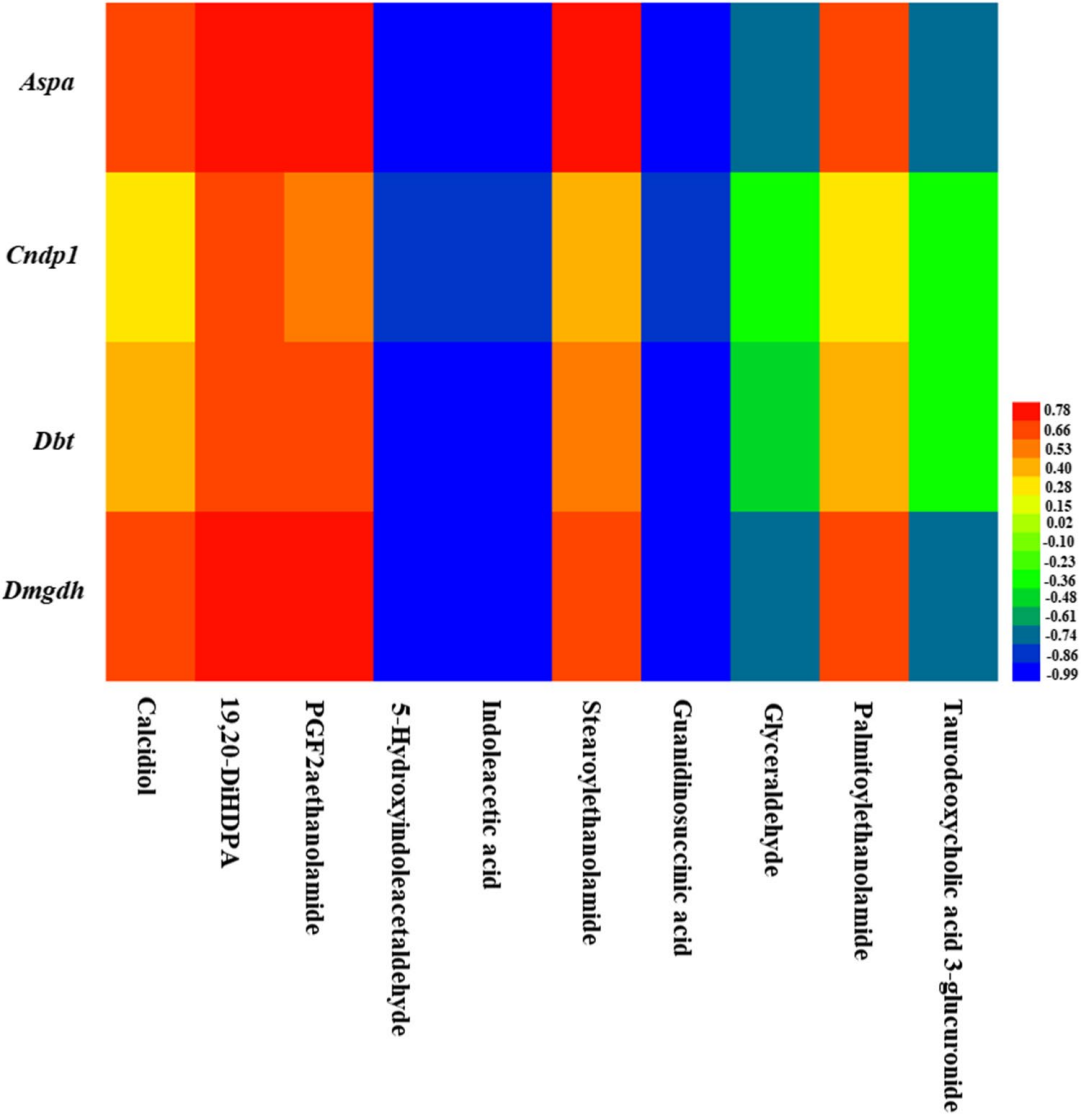
Heat map of correlation coefficients between differential genes and metabolites.

**Figure 4:**
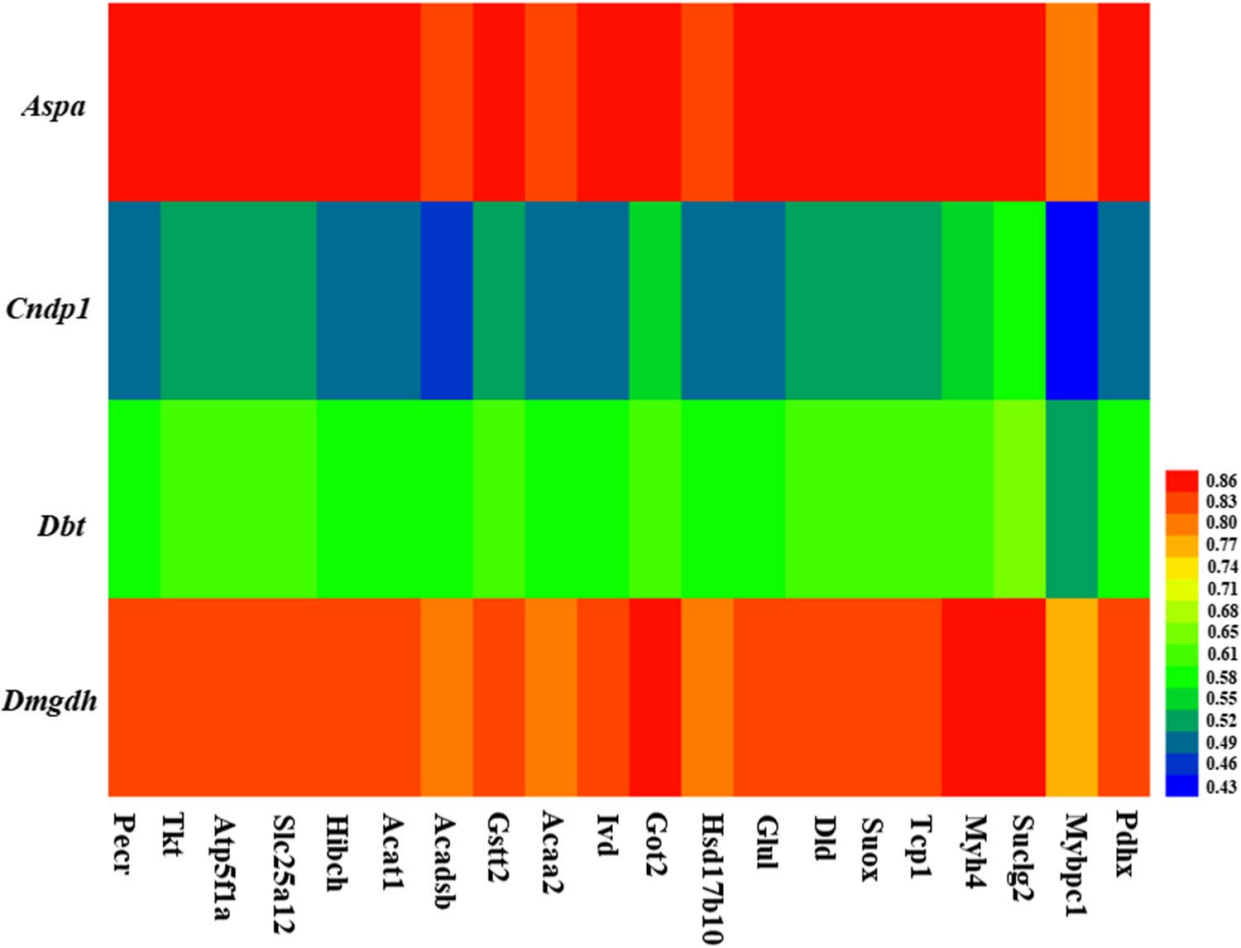
Heat map of correlation coefficients between differential genes and acetylated proteins.

**Figure 5:**
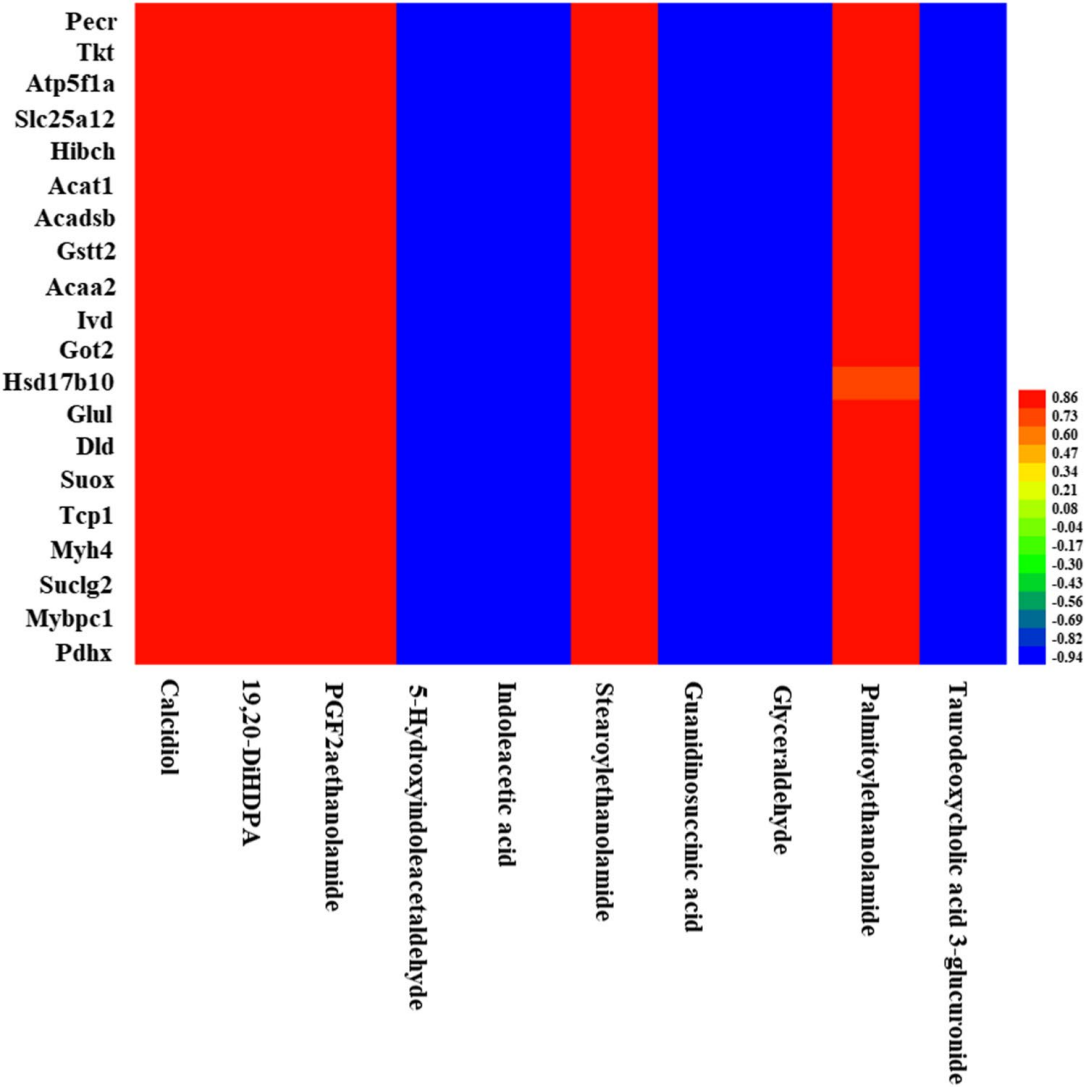
Heat map of correlation coefficients between differential metabolites and acetylated proteins.

***Aspa*** was highly positively correlated with calcidiol, 19,20-DiHDPA, PGF2a ethanolamide, stearoylethanolamide, palmitoylethanolamide, but highly negatively correlated with 5-hydroxyindoleacetaldehyde, indoleacetic acid, guanidinosuccinic acid, glyceraldehyde, and taurodeoxycholic acid 3-glucuronide (Fig. 3). ***Cndp1*** was highly positively correlated with 19,20-DiHDPA, PGF2aethanolamide and stearoylethanolamide, but highly negatively correlated with 5-hydroxyindole- acetaldehyde, indoleacetic acid and guanidinosuccinic acid (Fig. 3). ***Dbt*** was highly positively correlated with 19,20-DiHDPA, PGF2aethanolamide and stearoyl- ethanolamide, but highly negatively correlated with 5-hydroxyindoleacetaldehyde, indoleacetic acid, guanidinosuccinic acid and glyceraldehyde (Fig. 3). ***Dmgdh*** was highly positively correlated with calcidiol, 19,20-DiHDPA, PGF2aethanolamide, stearoylethanolamide and palmitoylethanolamide, but highly negatively correlated with 5-hydroxyindoleacetaldehyde, indoleacetic acid, guanidinosuccinic acid, glycer- aldehyde and taurodeoxycholic acid 3-glucuronide (Fig. 3). All the top 20 proteins with acetylation modification were highly positively correlated with ***Aspa***, ***Cndp1***, ***Dbt*** and ***Dmgdh*** (Fig. 4). All the top 20 proteins with acetylation modification were highly positively correlated with calcidiol, 19,20-DiHDPA, PGF2aethanolamide, stearoylethanolamide and palmitoylethanolamide, but highly negatively correlated with 5-hydroxyindoleacetaldehyde, indoleacetic acid, guanidinosuccinic acid, glycer- aldehyde and taurodeoxycholic acid 3-glucuronide (Fig. 5).

### Amino acid content in rat serum after fructose exposure

We further verified whether the changed trends of amino acids content in rat serum were consistent with that of the human population. As shown in Table 7, Trp, Arg, Ala, Asn, Pro, Tyr, bAla, Aad, bAib, Hyl, Hyp, EtN, PEtN and Ans significantly decreased while Abu significantly increased in rat serum after 6 months exposure of fructose. Among these significantly altered amino acids, the changed trends of 6 amino acids such as Arg, Tyr, Aad, Hyl, Hyp and PEtN were consistent with human, while the changed trends of 2 amino acids such as EtN and Ans were contrary to human (Table 7).

**Table 7:** Amino acid content in rat serum after fructose exposure.

Amino Acid	Control (μg/mL)	Fructose (μg/mL)	P	Trend
Ile	13.94 ± 2.09	14.96 ± 2.57	0.507	
Leu	18.88 ± 2.44	20.49 ± 3.65	0.432	
Lys	88.77 ± 6.53	70.85 ± 18.58	0.069	
Met	10.24 ± 1.19	8.79 ± 1.52	0.125	
Phe	16.70 ± 2.11	13.94 ± 2.47	0.087	
Thr	40.82 ± 5.78	34.94 ± 4.78	0.110	
Trp	34.16 ± 5.21	19.22 ± 4.39	0.001	**↓**
Val	21.52 ± 1.51	23.07 ± 3.49	0.385	
Arg	51.28 ± 8.25	36.35 ± 8.11	0.016	**↓**^**1**^
His	14.13 ± 2.00	12.66 ± 1.55	0.222	
Ala	52.48 ± 3.55	42.89 ± 8.30	0.039	**↓**
Asn	12.50 ± 0.95	10.17 ± 1.49	0.015	**↓**
Asp	7.30 ± 2.13	5.92 ± 2.21	0.338	
Cys	6.13 ± 1.37	4.98 ± 1.54	0.242	
Glu	20.39 ± 4.80	17.03 ± 4.06	0.259	
Gln	147.61 ± 8.70	130.92 ± 18.51	0.098	
Gly	34.71 ± 3.11	32.63 ± 4.21	0.395	
Pro	21.63 ± 1.92	16.60 ± 2.10	0.003	**↓**
Ser	37.30 ± 3.30	31.43 ± 5.42	0.066	
Tyr	19.27 ± 2.54	13.03 ± 2.34	0.002	**↓**^**1**^
bAla	1.10 ± 0.60	0.48 ± 0.09	0.043	**↓**
Abu	0.60 ± 0.06	1.08 ± 0.23	0.001	**↑**
GABA	0.04 ± 0.01	0.03 ± 0.01	0.156	
Aad	0.26 ± 0.03	0.20 ± 0.02	0.009	**↓**^**1**^
bAib	0.03 ± 0.01	0.01 ± 0.00	0.009	**↓**
Cit	15.41 ± 1.53	14.17 ± 2.32	0.340	
Hyl	0.23 ± 0.03	0.15 ± 0.04	0.003	**↓**^**1**^
Hyp	3.93 ± 0.51	2.82 ± 0.45	0.004	**↓**^**1**^
Sar	0.19 ± 0.03	0.22 ± 0.03	0.300	
Tau	40.18 ± 11.16	35.15 ± 6.91	0.411	
EtN	1.51 ± 0.22	1.08 ± 0.25	0.015	**↓**^**2**^
Cth	0.15 ± 0.03	0.12 ± 0.06	0.318	
Hcit	0.19 ± 0.05	0.16 ± 0.07	0.481	
3MHis	5.44 ± 1.23	4.22 ± 0.49	0.067	
1MHis	0.89 ± 0.18	0.89 ± 0.08	0.971	
PEtN	1.15 ± 0.41	0.69 ± 0.19	0.047	**↓**^**1**^
Car	1.07 ± 1.37	0.24 ± 0.18	0.207	
Ans	3.40 ± 2.30	0.83 ± 0.92	0.050	**↓**^**2**^
Asa	0.37 ± 0.08	0.32 ± 0.07	0.386	

^1^Consistent with the changes in human serum.

^2^Contrary to the changes in human serum.

## Discussion

Along with the changes of food consumption patterns toward high-calorie diets rich in sugar sweetened beverages and processed foods containing HFCS, fructose consumption has increased substantially during the last few decades^5^. Previous studies reported that the consumption of fructose is associated with intrahepatic lipid accumulation, hypertriglyceridemia and hyperuricemia^20,21^. Our recent study have shown that long-term exposure to common dose of fructose (the common soft drinks contain about 5% in common soft drinks) may lead to energy metabolic damage in adipose tissue and significant changes in serum important adipocytokines^22^. Fructose is absorbed in the small intestine and metabolized in the liver where it stimulates fructolysis, glycolysis, lipogenesis, and glucose production^20^. In current study, we found fasting blood glucose and triglycerides significantly increased in high fructose exposing individuals. However, the uric acid levels of high fructose exposing individuals did not significantly increased. Previous animal experiments shown that fructose could cause the development of hypertension and increase the heart rates^23^. In current study, we found fructose significantly increased the heart rates but had no significant effect on blood pressure in community residents.

AAs, the most common metabolites in body, are organic compounds that are derivatives of hydrocarbons, containing amino groups (–NH_2_) and carbonyl groups (-COOH). AAs can effectively reflect the health status of human. Especially in recent years, amino acids profile analysis in human biological fluids using modern analytical methods with the ability of simultaneous quantification of dozens of amino acids has aroused great interest among scientists^24^. In current study, we quantified 39 amino acids in the serum of high fructose exposing and control populations by UPLC-QqQMS based targeted metabolomics and found several amino acid biomarkers such as Asa, EtN, Asp, Glu, 1MHis, PEtN, Arg, Gln, GABA, aAd, Hyl and Cys for fructose exposure.

Gln, a nonessential amino acid (NEAA) which has many roles in cell metabolism, participating in tricarboxylic acid (TCA) cycle supplementation. Gln is transported into the mitochondrial matrix through the SLC1A5 variant and subsequently converted to glutamate (Glu). As a decomposition product of Gln, Glu plays a central role in NEAA metabolism because it is crucial for the biosynthesis of Ala, Asp, Pro, and Ser, which are in turn used for the biosynthesis of Asn, Arg, cysteine, and Gly^25^. Previous study have found that fructose reprogrammed cellular metabolic pathways to facilitate Gln breakdown^14^. Present study displayed Glu and Asp significantly elevated while Arg and Gln significantly decreased among individuals with high fructose exposure in the community. We did not find fructose had significant effect in population on other NEAAs synthesized from Glu, such as Ala, Asp, Pro, Ser, Asn and Gly.

Glu generats α-ketoglutarate which enters the TCA cycle and thereby affecting the energy metabolism of cells, through several enzymes such as glutamic-oxaloacetic transaminase 2 **(Got2)**, glutamic-pyruvate transaminase **(Gpt)** and phosphoserine aminotransferase 1 **(Psat1)**. Fructose did not seem to affect the expression of enzymes such as Got1, Psat1 and Gpt which are involved in the liver Gln metabolism pathway. However, the acetylated modification level of lysine at position 302 of **Got2** significantly increased after fructose exposure. Meanwhile, the acetylated modification level of **SLC25A1** and **SLC25A12**, transporters on mitochondrial membranes for citrate and Asp, also significantly increased after fructose exposure. The known function of **SLC25A1** consists of promoting the export of citrate or isocitrate from the mitochondria into the cytosol in exchange for malate. After entering the cytoplasm from mitochondria through SLC25A1, citric acid has several fundamental functions, on one side providing the source for fatty acids and sterol biosynthesis, and on the other, serving as an allosteric regulator for enzymes that control glycolysis, lipogenesis and gluconeogenesis^26^. Inhibition of **SLC25A1** could inhibit the growth of different tumor types^26,27^ and revert steatosis, glucose intolerance, and inflammation in preclinical models of nonalcoholic fatty liver disease/inflammatory steatohepatitis^28^. Acetylation of SLC25A1 increased citrate efflux in exchange with malate, to respond to the increasing cellular NAPDH demand^29^.

Aspartate-glutamate carrier isoform 1, encoded by *SLC25A12*, catalyzes an exchange between intramitochondrial Asp and cytosolic Glu plus a proton across the mitochondrial membrane, so supplying Asp to the cytosol^30^. SLC25A12 is silenced in normal liver. However, It can be activated in some tumor cells through histone acetylation modification^30^. The acetylated modification of SLC25A12 self in liver is still rarely reported. Further research on the function of acetylated modification of SLC25A12 is needed.

Aspartoacylase (***Aspa****)* is a regulated nuclear-cytoplasmic enzyme which catalyzes deacetylation of *N*-acetyl-l-aspartate^31^. Expression of ***Aspa*** in liver downregulated by more than 2 times after fructose exposure. Low expression of ***Aspa*** in liver may be related to the responsive regulation of high levels of circulating Asp.

Notably, Asp and Asa are common components of the aspartate-arginosuccinate shunt, a set of transformations connecting the TCA cycle with the urea cycle^32^. Fructose led to a significant increase in both Asp and Asa in community residents, which may be related to the activation of aspartate-arginosuccinate shunt. Guanidinosuccinic acid, a representative guanidino compounds that is produced by the urea cycle and transamidination from Arg, significantly decreased after fructose exposure in animal liver, which is another evidence that fructose affects the Urea cycle.

Both aAd and Hyl are Lys derivatives. Serum free Lys did not changed significantly in high fructose exposing population, however, aAd and Hyl decreased significantly in high fructose exposing population. 1MHis is a precursor of anserine (β-alanine-1-methyl histidine, Ans) in muscle. It is generated due to the transmethylation of S-adenosyl methionine to His. The changes of 1MHis, aAd and Hyl indicated fructose affected both the metabolism in bone and muscle.

Both EtN and PEtN are raw materials for synthesis of phosphatidylethanolamine, which plays a very important role in cell morphology, metabolic regulation, signal transduction, and various physiological functions of cells. Ser can be metabolized into EtN and PEtN via serine decarboxylation. However, we did not find Ser changed significantly in high fructose exposing population. Significant increase in EtN and significant decrease in PEtN in high fructose exposing population indicated fructose significantly interfered with effective utilization of EtN.

Blood calcidiol (25-hydroxyvitamin D) can effectively reflect the lack of vitamin D in the body. Previous research reported dietary fructose inhibited intestinal calcium absorption and induced vitamin D insufficiency in chronic kidney disease^33^. We speculate that fructose exposure may cause compensatory increase of hepatic synthesis of calcidiol. However, further evidence is needed. PGF2a-ethanolamide, stearoylethanolamide and palmitoylethanolamide are *N*-acylethanol-amines which were reported to be related to hepatic cancer, steatosis and cirrhosis^34^. The serum EtN is an important source for liver synthesis of *N*-acetylethanolamines. The increase of *N*-acetylethanolamines in liver could explain why serum EtN increased. Taurodeoxycholic acid 3-glucuronide is a relatively new metabolite, which is rarely reported, and may serve as a new marker for liver response to fructose exposure.

The liver is the most important organ for metabolizing amino acids. Amino acid, as one of the three major nutrients in the body (the other two are sugar and lipid), long-term exposure to fructose had led to significant changes in the metabolomics of mouse liver, indicating that fructose is highly likely to alter the amino acid metabolism of the liver. The significant increase of *N*-acetylethanolamines provided a valuable clue to the future study of the mechanism of metabolic damage caused by fructose in the body.

***Cndp1*** is responsible for encoding carnosine dipeptidase which decomposes Car (β-alanyl-l-histidine) into Ala and His. In present study, fasting serum Car did not changed significantly in high fructose exposing group. However, PCR array displayed 6 months fructose exposure caused liver expressed higher level of ***Cndp1*** in rats. The expression of ***Dbt***, which encodes the core component of branched-chain alphaketo acid dehydrogenase complex,was similar to ***Cndp1*** in liver. We did not observe any changes in **Dbt**’s substrates such as Leu, Ile and Val in serum. These inconsistent phenomena may be due to species differences.

***Dmgdh*** encodes a mammalian flavin mitochondrial enzyme, which catalyzes the oxidative demethylation of dimethylglycine^35^. ***Dmgdh*** plays an important role in the utilization of methyl groups derived from choline. **Sar**, a product of the oxidative demethylation of dimethylglycine, is known as an important intermediate in one-carbon metabolism. In present study, the expression of ***Dmgdh*** decreased significantly in rat liver after fructose exposure. And in high fructose exposing population, **Sar** also displayed a downtrend (P < 0.05, corrected P value = 0.10).

Additionally, we found the effect of fructose on amino acid metabolism in the human population was different from that in the rats, of which the main reason may be species differences. However, we still found the decrease in rats of 6 amino acids such as Arg, Tyr, Aad, Hyl, Hyp and PEtN was consistent with human. These findings indicated the decrease in Arg, Tyr, Aad, Hyl, Hyp and PEtN induced by fructose exposure may be conserved among different species.

Fructose can enhance glycolysis, promoting processes to store rather than use energy. Therefore, fructose once assisted human ancestors in surviving under extreme conditions such as severe climate change, asteroid impacts, food shortage and droughts^36^. However, it is precisely due to this unique metabolic characteristic that fructose is more likely to induce glucose and lipid metabolism damage. Amino acids metabolism is crucial for health, not only because amino acids are the raw materials for synthesizing proteins in the body, but also because amino acid metabolism is an important link with glucose and lipid metabolism. However, less attention has previously been paid to the impact of fructose on amino acid metabolism. This study can provide reliable supports for comprehensive understanding the interference of fructose on amino acid metabolism in the body.

## Conclusion

Population based amino acid targeted metabolomics study showed that people with high level of fructose had higher levels of Asa, EtN, Asp, and Glu, whereas they had lower levels of 1MHis, PEtN, Arg, Gln, GABA, Aad, Hyl and Cys. The further mechanism study in rats model displayed amino acid metabolic genes of ***Aspa***, ***Cndp1*, *Dbt***, ***Dmgdh,*** and toxic metabolites such as ***N*-acetylethanolamines** accumulation, interference of urea cycle, as well as acetylated modification of key enzymes in **glutamine metabolic network** and **glutamine derived NEAAs synthesis** pathway in liver may play important roles in fructose caused reprogramming in amino acid metabolism.

### Supplementary Information

Supplementary Table 1.

Supplementary Table 2.

Supplementary Table 3.

Supplementary Table 4.

## Data Availability

The data analyzed during current study are available from the corresponding author upon reasonable request. Contact Mu Wang (wangmu2009@163.com) for additional information regarding data access.
