# Supplementary material for: The effect of fructose exposure on amino acid metabolism among Chinese community residents and its possible multi-omics mechanisms
Source: Sci Rep. 2023 Dec 19;13:22704. doi: 10.1038/s41598-023-50069-5 (PMC10733306; doi:10.1038/s41598-023-50069-5)
Supplement: Supplementary file 1 — Supplementary Table 1. [file 41598_2023_50069_MOESM1_ESM.docx]

**Supplementary table 1 MRM parameters of 39 amino acids in serum**

| Compound | Parent(m/z) | Daughter(m/z) | Dwell(s) | Cone(v) | Collision(v) |
| --- | --- | --- | --- | --- | --- |
| His | 163.76 | 110.05 | 0.001 | 24 | 20 |
| 1MHis | 170.62 | 124.02 | 0.001 | 23 | 20 |
| 3MHis | 170.87 | 95.88 | 0.01 | 28 | 17 |
| Arg | 173.19 | 171.02 | 0.001 | 26 | 25 |
| Car | 199.21 | 171.06 | 0.01 | 26 | 17 |
| Ans | 206.26 | 171.06 | 0.01 | 29 | 13 |
| Asa | 231.07 | 171.04 | 0.01 | 24 | 14 |
| EtN | 232.0957 | 171.0983 | 0.001 | 38 | 35 |
| Lys | 317.1503 | 171.0942 | 0.001 | 38 | 20 |
| Gly | 246.0319 | 171.0716 | 0.001 | 36 | 40 |
| Hyl | 333.2234 | 171.0766 | 0.001 | 36 | 17 |
| Sar | 260.0958 | 171.0838 | 0.001 | 34 | 30 |
| bAla | 260.0958 | 171.1024 | 0.001 | 34 | 18 |
| Ala | 260.0958 | 171.0838 | 0.001 | 34 | 50 |
| GABA | 274.0958 | 171.0952 | 0.001 | 36 | 30 |
| bAib | 274.0958 | 171.1011 | 0.001 | 38 | 20 |
| Abu | 274.0958 | 171.0952 | 0.001 | 36 | 40 |
| Ser | 276.0958 | 171.0868 | 0.001 | 34 | 45 |
| Cth | 393.2873 | 171.04 | 0.001 | 33 | 19 |
| Pro | 286.1596 | 171.0797 | 0.001 | 32 | 50 |
| Val | 288.2873 | 171.0706 | 0.001 | 42 | 55 |
| Thr | 290.1596 | 171.0985 | 0.001 | 36 | 40 |
| Cys | 411.2 | 171.09 | 0.001 | 30 | 15 |
| Tau | 296.0958 | 171.0742 | 0.001 | 26 | 14 |
| Hyp | 302.1596 | 171.0844 | 0.001 | 40 | 25 |
| Leu | 302.1596 | 171.0844 | 0.028 | 40 | 60 |
| Ile | 302.1596 | 171.0883 | 0.028 | 42 | 24 |
| Asn | 303.0958 | 171.0951 | 0.001 | 38 | 40 |
| Asp | 304.2234 | 171.0747 | 0.001 | 20 | 40 |
| PEtN | 312.0958 | 171.0774 | 0.001 | 25 | 30 |
| Gln | 317.1503 | 171.0942 | 0.001 | 38 | 60 |
| Glu | 318.1596 | 171.1504 | 0.001 | 45 | 50 |
| Met | 320.1596 | 171.0783 | 0.001 | 42 | 45 |
| Aad | 332.1596 | 171.0664 | 0.001 | 35 | 20 |
| Phe | 336.1596 | 171.0811 | 0.028 | 44 | 55 |
| Cit | 346.2234 | 171.1387 | 0.01 | 33 | 20 |
| Tyr | 352.2234 | 171.0848 | 0.001 | 36 | 45 |
| Hcit | 360.2234 | 171.0860 | 0.01 | 37 | 24 |
| Trp | 375.2234 | 171.0770 | 0.028 | 42 | 50 |

**Abbr.**: Histidine (His); 1-Methyl-L-histidine (1MHis); 3-Methyl-L-histidine (3MHis); Hydroxy- proline (Hyp); Asparagine (Asn); Phosphorylethanolamine (PEtN); Arginine (Arg); Carnosine (Car); Anserine (Ans); Argininosuccinic acid (Asa); Serine (Ser); Taurine (Tau); Glutamine (Gln); Ethanolamine (EtN); Glycine (Gly); Sarcosine (Sar); beta-Alanine (bAla); Threonine (Thr); Aspartic acid (Asp); Glutamic acid (Glu); Citrulline (Cit); Alanine (Ala); gamma-Aminobutyric acid (GABA); Aminoisobutyric acid (bAib); Proline (Pro); Aminoadipic acid (aAd); 5-Hydroxylysine (Hyl); Homocitrulline (Hcit); 2-Aminobutyric acid (Abu); Valine (Val); Methionine (Met); Tyrosine (Tyr); Cystathionine (Cth); Cystine (Cys); Leucine (Leu); Isoleucine (Ile); Phenylalanine (Phe); Tryptophan (Trp); Lysine (Lys)
