# Supplementary material for: The effect of fructose exposure on amino acid metabolism among Chinese community residents and its possible multi-omics mechanisms
Source: Sci Rep. 2023 Dec 19;13:22704. doi: 10.1038/s41598-023-50069-5 (PMC10733306; doi:10.1038/s41598-023-50069-5)
Supplement: Supplementary file 2 — Supplementary Table 2. [file 41598_2023_50069_MOESM2_ESM.docx]

**Supplementary table 2 The list of 84 amino acid metabolism genes**

| **Position** | **Unigene** | **Refseq** | **Symbol** | **Description** | **RT2 Catalog** |
| --- | --- | --- | --- | --- | --- |
| A01 | Rn.11133 | NM_017193 | *Aadat* | Aminoadipate aminotransferase | PPR45252A |
| A02 | Rn.17217 | NM_001106798 | *Aasdhppt* | Aminoadipate-semialdehyde dehydrogenase-phosphopantetheinyl transferase | [PPR46439A](https://geneglobe.qiagen.com/search?cat=&q=PPR46439A) |
| A03 | Rn.198671 | NM_001100963 | *Aass* | Aminoadipate-semialdehyde synthase | [PPR56448B](https://geneglobe.qiagen.com/search?cat=&q=PPR56448B) |
| A04 | Rn.10090 | NM_031003 | *Abat* | 4-aminobutyrate aminotransferase | [PPR06756A](https://geneglobe.qiagen.com/search?cat=&q=PPR06756A) |
| A05 | Rn.54493 | NM_022935 | *Aoc1* | Amiloride binding protein 1 (amine oxidase, copper-containing) | [PPR50814A](https://geneglobe.qiagen.com/search?cat=&q=PPR50814A) |
| A06 | Rn.3786 | NM_130433 | *Acaa2* | Acetyl-Coenzyme A acyltransferase 2 | [PPR43229A](https://geneglobe.qiagen.com/search?cat=&q=PPR43229A) |
| A07 | Rn.6302 | NM_016986 | *Acadm* | Acyl-Coenzyme A dehydrogenase, C-4 to C-12 straight chain | [PPR43572A](https://geneglobe.qiagen.com/search?cat=&q=PPR43572A) |
| A08 | Rn.1167 | NM_022512 | *Acads* | Acyl-Coenzyme A dehydrogenase, C-2 to C-3 short chain | [PPR42424A](https://geneglobe.qiagen.com/search?cat=&q=PPR42424A) |
| A09 | Rn.44423 | NM_013084 | *Acadsb* | Acyl-Coenzyme A dehydrogenase, short/branched chain | [PPR49766A](https://geneglobe.qiagen.com/search?cat=&q=PPR49766A) |
| A10 | Rn.203063 | NM_001006995 | *Acat2* | Acetyl-Coenzyme A acetyltransferase 3 | [PPR59638A](https://geneglobe.qiagen.com/search?cat=&q=PPR59638A) |
| A11 | Rn.137460 | NM_001106475 | *Adh6a* | Alcohol dehydrogenase 6A (class V) | [PPR52199A](https://geneglobe.qiagen.com/search?cat=&q=PPR52199A) |
| A12 | Rn.23276 | NM_001130503 | *Adsl* | Adenylosuccinate lyase | [PPR55117A](https://geneglobe.qiagen.com/search?cat=&q=PPR55117A) |
| B01 | Rn.9047 | NM_001105975 | *Adss* | Adenylosuccinate synthase | [PPR44263A](https://geneglobe.qiagen.com/search?cat=&q=PPR44263A) |
| B02 | Rn.9931 | NM_030656 | *Agxt* | Alanine-glyoxylate aminotransferase | [PPR44567A](https://geneglobe.qiagen.com/search?cat=&q=PPR44567A) |
| B03 | Rn.97126 | NM_024484 | *Alas1* | Aminolevulinate, delta-, synthase 1 | [PPR53477B](https://geneglobe.qiagen.com/search?cat=&q=PPR53477B) |
| B04 | Rn.101781 | NM_032416 | *Aldh2* | Aldehyde dehydrogenase 2 family (mitochondrial) | [PPR54702F](https://geneglobe.qiagen.com/search?cat=&q=PPR54702F) |
| B05 | Rn.162510 | NM_001006998 | *Aldh3b1* | Aldehyde dehydrogenase 3 family, member B1 | [PPR48182A](https://geneglobe.qiagen.com/search?cat=&q=PPR48182A) |
| B06 | Rn.10070 | NM_022851 | *Aldh5a1* | Aldehyde dehydrogenase 5 family, member A1 | [PPR44654A](https://geneglobe.qiagen.com/search?cat=&q=PPR44654A) |
| B07 | Rn.2098 | NM_031057 | *Aldh6a1* | Aldehyde dehydrogenase 6 family, member A1 | [PPR42668C](https://geneglobe.qiagen.com/search?cat=&q=PPR42668C) |
| B08 | Rn.162376 | NM_001014004 | *Amt* | Aminomethyltransferase | [PPR47809B](https://geneglobe.qiagen.com/search?cat=&q=PPR47809B) |
| B09 | Rn.198327 | NM_031582 | *Aoc3* | Amine oxidase, copper containing 3 (vascular adhesion protein 1) | [PPR50952A](https://geneglobe.qiagen.com/search?cat=&q=PPR50952A) |
| B10 | Rn.86973 | NM_001107689 | *Ash1l* | Ash1 (absent, small, or homeotic)-like (Drosophila) | [PPR51927B](https://geneglobe.qiagen.com/search?cat=&q=PPR51927B) |
| B11 | Rn.11172 | NM_013079 | *Asns* | Asparagine synthetase | [PPR45274A](https://geneglobe.qiagen.com/search?cat=&q=PPR45274A) |
| B12 | Rn.21677 | NM_024399 | *Aspa* | Aspartoacylase | [PPR47195A](https://geneglobe.qiagen.com/search?cat=&q=PPR47195A) |
| C01 | Rn.16333 | NM_022629 | *Bbox1* | Butyrobetaine (gamma), 2-oxoglutarate dioxygenase (gamma-butyrobetaine hydroxylase) 1 | [PPR50276A](https://geneglobe.qiagen.com/search?cat=&q=PPR50276A) |
| C02 | Rn.8273 | NM_017253 | *Bcat1* | Branched chain aminotransferase 1, cytosolic | [PPR44047A](https://geneglobe.qiagen.com/search?cat=&q=PPR44047A) |
| C03 | Rn.15623 | NM_019267 | *Bckdhb* | Branched chain keto acid dehydrogenase E1, beta polypeptide | [PPR46140A](https://geneglobe.qiagen.com/search?cat=&q=PPR46140A) |
| C04 | Rn.11406 | NM_030850 | *Bhmt* | Betaine-homocysteine methyltransferase | [PPR45418A](https://geneglobe.qiagen.com/search?cat=&q=PPR45418A) |
| C05 | Rn.22857 | NM_198731 | *Chdh* | Choline dehydrogenase | [PPR55499A](https://geneglobe.qiagen.com/search?cat=&q=PPR55499A) |
| C06 | Rn.15548 | NM_001007687 | *Cndp1* | Carnosine dipeptidase 1 (metallopeptidase M20 family) | [PPR59610A](https://geneglobe.qiagen.com/search?cat=&q=PPR59610A) |
| C07 | Rn.220 | NM_012531 | *Comt* | Catechol-O-methyltransferase | [PPR06789A](https://geneglobe.qiagen.com/search?cat=&q=PPR06789A) |
| C08 | Rn.81058 | NM_053626 | *Dao* | D-amino-acid oxidase | [PPR52255A](https://geneglobe.qiagen.com/search?cat=&q=PPR52255A) |
| C09 | Rn.87166 | NM_013158 | *Dbh* | Dopamine beta-hydroxylase (dopamine beta-monooxygenase) | [PPR52652A](https://geneglobe.qiagen.com/search?cat=&q=PPR52652A) |
| C10 | Rn.198610 | NM_053312 | *Dbt* | Dihydrolipoamide branched chain transacylase E2 | [PPR53153B](https://geneglobe.qiagen.com/search?cat=&q=PPR53153B) |
| C11 | Rn.11064 | NM_012545 | *Ddc* | Dopa decarboxylase (aromatic L-amino acid decarboxylase) | [PPR45208A](https://geneglobe.qiagen.com/search?cat=&q=PPR45208A) |
| C12 | Rn.86962 | NM_199385 | *Dld* | Dihydrolipoamide dehydrogenase | [PPR52634A](https://geneglobe.qiagen.com/search?cat=&q=PPR52634A) |
| D01 | Rn.99702 | NM_001006981 | *Dlst* | Dihydrolipoamide S-succinyltransferase (E2 component of 2-oxo-glutarate complex) | [PPR59415A](https://geneglobe.qiagen.com/search?cat=&q=PPR59415A) |
| D02 | Rn.3646 | NM_139102 | *Dmgdh* | Dimethylglycine dehydrogenase | [PPR43174A](https://geneglobe.qiagen.com/search?cat=&q=PPR43174A) |
| D03 | Rn.6847 | NM_078623 | *Echs1* | Enoyl Coenzyme A hydratase, short chain, 1, mitochondrial | [PPR43709A](https://geneglobe.qiagen.com/search?cat=&q=PPR43709A) |
| D04 | Rn.9195 | NM_017181 | *Fah* | Fumarylacetoacetate hydrolase | [PPR44293A](https://geneglobe.qiagen.com/search?cat=&q=PPR44293A) |
| D05 | Rn.20140 | NM_053567 | *Ftcd* | Formiminotransferase cyclodeaminase | [PPR46972A](https://geneglobe.qiagen.com/search?cat=&q=PPR46972A) |
| D06 | Rn.29951 | NM_012563 | *Gad2* | Glutamate decarboxylase 2 | [PPR48349A](https://geneglobe.qiagen.com/search?cat=&q=PPR48349A) |
| D07 | Rn.43940 | NM_001024277 | *Gcat* | Glycine C-acetyltransferase (2-amino-3-ketobutyrate-coenzyme A ligase) | [PPR49666A](https://geneglobe.qiagen.com/search?cat=&q=PPR49666A) |
| D08 | Rn.99039 | NM_001108896 | *Gcdh* | Glutaryl-Coenzyme A dehydrogenase | [PPR44208A](https://geneglobe.qiagen.com/search?cat=&q=PPR44208A) |
| D09 | Rn.17101 | NM_001107583 | *Gldc* | Glycine dehydrogenase (decarboxylating) | [PPR46414A](https://geneglobe.qiagen.com/search?cat=&q=PPR46414A) |
| D10 | Rn.11142 | NM_017084 | *Gnmt* | Glycine N-methyltransferase | [PPR45257A](https://geneglobe.qiagen.com/search?cat=&q=PPR45257A) |
| D11 | Rn.5819 | NM_012571 | *Got1* | Glutamic-oxaloacetic transaminase 1, soluble (aspartate aminotransferase 1) | [PPR43460A](https://geneglobe.qiagen.com/search?cat=&q=PPR43460A) |
| D12 | Rn.6318 | NM_031039 | *Gpt* | Glutamic-pyruvate transaminase (alanine aminotransferase) | [PPR43579A](https://geneglobe.qiagen.com/search?cat=&q=PPR43579A) |
| E01 | Rn.92789 | NM_057186 | *Hadh* | Hydroxyacyl-Coenzyme A dehydrogenase | [PPR53155A](https://geneglobe.qiagen.com/search?cat=&q=PPR53155A) |
| E02 | Rn.11253 | NM_133618 | *Hadhb* | Hydroxyacyl-Coenzyme A dehydrogenase/3-ketoacyl-Coenzyme A thiolase/enoyl-Coenzyme A hydratase (trifunctional protein), beta subunit | [PPR45326A](https://geneglobe.qiagen.com/search?cat=&q=PPR45326A) |
| E03 | Rn.48653 | NM_017016 | *Hdc* | Histidine decarboxylase | [PPR50228A](https://geneglobe.qiagen.com/search?cat=&q=PPR50228A) |
| E04 | Rn.24631 | NM_001012145 | *Hgd* | Homogentisate 1, 2-dioxygenase | [PPR59483B](https://geneglobe.qiagen.com/search?cat=&q=PPR59483B) |
| E05 | Rn.73 | NM_022243 | *Hibadh* | 3-hydroxyisobutyrate dehydrogenase | [PPR42257A](https://geneglobe.qiagen.com/search?cat=&q=PPR42257A) |
| E06 | Rn.8745 | NM_001013112 | *Hibch* | 3-hydroxyisobutyryl-Coenzyme A hydrolase | [PPR44186A](https://geneglobe.qiagen.com/search?cat=&q=PPR44186A) |
| E07 | Rn.13145 | NM_031044 | *Hnmt* | Histamine N-methyltransferase | [PPR45784A](https://geneglobe.qiagen.com/search?cat=&q=PPR45784A) |
| E08 | Rn.3664 | NM_017233 | *Hpd* | 4-hydroxyphenylpyruvate dioxygenase | [PPR43182A](https://geneglobe.qiagen.com/search?cat=&q=PPR43182A) |
| E09 | Rn.2700 | NM_031682 | *Hsd17b10* | Hydroxysteroid (17-beta) dehydrogenase 10 | [PPR42824A](https://geneglobe.qiagen.com/search?cat=&q=PPR42824A) |
| E10 | Rn.61745 | NM_001100572 | *Iars* | Isoleucyl-tRNA synthetase | [PPR42283A](https://geneglobe.qiagen.com/search?cat=&q=PPR42283A) |
| E11 | Rn.17983 | NM_001011956 | *Lcmt2* | Leucine carboxyl methyltransferase 2 | [PPR46561A](https://geneglobe.qiagen.com/search?cat=&q=PPR46561A) |
| E12 | Rn.224544 | NM_033653 | *Maoa* | Monoamine oxidase A | [PPR46359B](https://geneglobe.qiagen.com/search?cat=&q=PPR46359B) |
| F01 | Rn.3420 | NM_001106341 | *Mcee* | Methylmalonyl CoA epimerase | [PPR43091A](https://geneglobe.qiagen.com/search?cat=&q=PPR43091A) |
| F02 | Rn.2661 | NM_031051 | *Mif* | Macrophage migration inhibitory factor | [PPR42812B](https://geneglobe.qiagen.com/search?cat=&q=PPR42812B) |
| F03 | N/A | XM_001067239 | *Mmut* | Methylmalonyl-CoA mutase | [PPR64881B](https://geneglobe.qiagen.com/search?cat=&q=PPR64881B) |
| F04 | Rn.38202 | NM_001106062 | *Ogdhl* | Oxoglutarate dehydrogenase-like | [PPR49049A](https://geneglobe.qiagen.com/search?cat=&q=PPR49049A) |
| F05 | Rn.1652 | NM_012619 | *Pah* | Phenylalanine hydroxylase | [PPR42550B](https://geneglobe.qiagen.com/search?cat=&q=PPR42550B) |
| F06 | Rn.6033 | NM_019330 | *Pcca* | Propionyl-coenzyme A carboxylase, alpha polypeptide | [PPR43498B](https://geneglobe.qiagen.com/search?cat=&q=PPR43498B) |
| F07 | Rn.11126 | NM_053994 | *Pdha2* | Pyruvate dehydrogenase (lipoamide) alpha 2 | [PPR45246A](https://geneglobe.qiagen.com/search?cat=&q=PPR45246A) |
| F08 | Rn.6872 | NM_031620 | *Phgdh* | Phosphoglycerate dehydrogenase | [PPR43715A](https://geneglobe.qiagen.com/search?cat=&q=PPR43715A) |
| F09 | Rn.163169 | NM_001012009 | *Pipox* | Pipecolic acid oxidase | [PPR59719B](https://geneglobe.qiagen.com/search?cat=&q=PPR59719B) |
| F10 | Rn.90152 | NM_178101 | *Plod3* | Procollagen-lysine, 2-oxoglutarate 5-dioxygenase 3 | [PPR52816C](https://geneglobe.qiagen.com/search?cat=&q=PPR52816C) |
| F11 | Rn.42 | NM_053576 | *Prdx6* | Peroxiredoxin 6 | [PPR42245A](https://geneglobe.qiagen.com/search?cat=&q=PPR42245A) |
| F12 | Rn.100813 | NM_198738 | *Psat1* | Phosphoserine aminotransferase 1 | [PPR54382A](https://geneglobe.qiagen.com/search?cat=&q=PPR54382A) |
| G01 | Rn.8734 | NM_001009679 | *Psph* | Phosphoserine phosphatase | [PPR44182A](https://geneglobe.qiagen.com/search?cat=&q=PPR44182A) |
| G02 | Rn.89832 | NM_053664 | *Sardh* | Sarcosine dehydrogenase | [PPR52795B](https://geneglobe.qiagen.com/search?cat=&q=PPR52795B) |
| G03 | Rn.9918 | NM_053962 | *Sds* | Serine dehydratase | [PPR44559A](https://geneglobe.qiagen.com/search?cat=&q=PPR44559A) |
| G04 | Rn.9214 | NM_001008322 | *Shmt2* | Serine hydroxymethyltransferase 2 (mitochondrial) | [PPR44295A](https://geneglobe.qiagen.com/search?cat=&q=PPR44295A) |
| G05 | Rn.220332 | NM_198757 | *Srr* | Serine racemase | [PPR49491A](https://geneglobe.qiagen.com/search?cat=&q=PPR49491A) |
| G06 | Rn.11082 | NM_012740 | *Th* | Tyrosine hydroxylase | [PPR45220F](https://geneglobe.qiagen.com/search?cat=&q=PPR45220F) |
| G07 | Rn.3607 | NM_133387 | *Tmlhe* | Trimethyllysine hydroxylase, epsilon | [PPR43160A](https://geneglobe.qiagen.com/search?cat=&q=PPR43160A) |
| G08 | Rn.233736 | NM_019353 | *Tpo* | Thyroid peroxidase | [PPR52879B](https://geneglobe.qiagen.com/search?cat=&q=PPR52879B) |
| G09 | Rn.38928 | NM_001107535 | *Tyr* | Tyrosinase | [PPR49133A](https://geneglobe.qiagen.com/search?cat=&q=PPR49133A) |
| G10 | Rn.40774 | NM_001106664 | *Tyrp1* | Tyrosinase-related protein 1 | [PPR49317A](https://geneglobe.qiagen.com/search?cat=&q=PPR49317A) |
| G11 | Rn.43153 | NM_213563 | *Vars2* | Valyl-tRNA synthetase 2, mitochondrial (putative) | [PPR45615B](https://geneglobe.qiagen.com/search?cat=&q=PPR45615B) |
| G12 | Rn.163205 | NM_001135743 | *Wbscr22* | Williams Beuren syndrome chromosome region 22 | [PPR53696B](https://geneglobe.qiagen.com/search?cat=&q=PPR53696B) |
| H01 | Rn.94978 | NM_031144 | *Actb* | Actin, beta | [PPR06570C](https://geneglobe.qiagen.com/search?cat=&q=PPR06570C) |
| H02 | Rn.1868 | NM_012512 | *B2m* | Beta-2 microglobulin | [PPR42607A](https://geneglobe.qiagen.com/search?cat=&q=PPR42607A) |
| H03 | Rn.47 | NM_012583 | *Hprt1* | Hypoxanthine phosphoribosyltransferase 1 | [PPR42247F](https://geneglobe.qiagen.com/search?cat=&q=PPR42247F) |
| H04 | Rn.107896 | NM_017025 | *Ldha* | Lactate dehydrogenase A | [PPR56603B](https://geneglobe.qiagen.com/search?cat=&q=PPR56603B) |
| H05 | Rn.973 | NM_001007604 | *Rplp1* | Ribosomal protein, large, P1 | [PPR42363C](https://geneglobe.qiagen.com/search?cat=&q=PPR42363C) |
| H06 | N/A | U26919 | RGDC | Rat Genomic DNA Contamination | [PPR63338A](https://geneglobe.qiagen.com/search?cat=&q=PPR63338A) |
| H07 | N/A | SA_00104 | RTC | Reverse Transcription Control | [PPX63340A](https://geneglobe.qiagen.com/search?cat=&q=PPX63340A) |
| H08 | N/A | SA_00104 | RTC | Reverse Transcription Control | [PPX63340A](https://geneglobe.qiagen.com/search?cat=&q=PPX63340A) |
| H09 | N/A | SA_00104 | RTC | Reverse Transcription Control | [PPX63340A](https://geneglobe.qiagen.com/search?cat=&q=PPX63340A) |
| H10 | N/A | SA_00103 | PPC | Positive PCR Control |  |
| H11 | N/A | SA_00103 | PPC | Positive PCR Control |  |
| H12 | N/A | SA_00103 | PPC | Positive PCR Control |  |
