# Supplementary material for: The effect of fructose exposure on amino acid metabolism among Chinese community residents and its possible multi-omics mechanisms
Source: Sci Rep. 2023 Dec 19;13:22704. doi: 10.1038/s41598-023-50069-5 (PMC10733306; doi:10.1038/s41598-023-50069-5)
Supplement: Supplementary file 3 — Supplementary Table 3. [file 41598_2023_50069_MOESM3_ESM.docx]

**Supplementary table 3 Amino acid metabolic difference between high fructose exposure male population in the larger quartile and the accurately matched male control**

|  | **Control** | **High fructose concentration group** | **P value** | **Corrected P value^#^** |
| --- | --- | --- | --- | --- |
| **Mean/median of fructose (μg/mL)** | 0 | 361.27/122.51 |  |  |
| **The highest/lowest level of fructose (μg/mL)** | 0 | 1478.72/41.29 |  |  |
| **Age** | 68.63±9.62^a^ | 68.66±9.69^a^ | 0.980 |  |
| **Male** | 104 | 104 |  |  |
| **Diabetes (n)** | 15 | 26 | 0.060 |  |
| **Uric acid (μMol.L^-1^)** | 354.11±73.38^a^ | 355.92±76.36^a^ | 0.860 |  |
| **Height (cm)** | 166.03±5.97^a^ | 165.08±5.93^a^ | 0.260 |  |
| **Weight (Kg)** | 65.49±10.54^a^ | 64.97±9.90^a^ | 0.720 |  |
| **Waist Circumference (cm)** | 84.54±10.79^a^ | 84.50±9.76^a^ | 0.980 |  |
| **BMI (kg/cm2)** | 23.69±3.20^a^ | 23.80±3.05^a^ | 0.810 |  |
| **Left Systolic Blood Pressure (mm/Hg)** | 138.25±18.81^a^ | 140.71±17.84^a^ | 0.340 |  |
| **Left Diastolic Blood Pressure (mm/Hg)** | 75.78±8.04^a^ | 75.88±7.47^a^ | 0.930 |  |
| **Right Systolic Blood Pressure (mm/Hg)** | 141.51±18.54^a^ | 143.90±17.39^a^ | 0.350 |  |
| **Right Diastolic Blood Pressure (mm/Hg)** | 79.14±7.59^a^ | 79.32±7.38^a^ | 0.860 |  |
| **Hemoglobin (g/L)** | 139.51±16.35^a^ | 141.24±12.26^a^ | 0.400 |  |
| **White Blood Cells (×10**9/L) | 6.86±1.61^a^ | 6.90±1.86^a^ | 0.870 |  |
| **Platelet (×10**9/**L)** | 216.74±177.26^a^ | 195.04±55.45^a^ | 0.240 |  |
| **Fasting Blood Glucose (mmol/L)** | 6.32±1.68^a^ | 7.35±2.73^a^ | 0.001 | 0.013 |
| **Serum ALT (U/L)** | 24.44±20.23^a^ | 24.43±14.87^a^ | 0.990 |  |
| **Serum AST (U/L)** | 23.94±13.84^a^ | 23.08±12.53^a^ | 0.650 |  |
| **Total Bilirubin (μMol/L)** | 15.38±5.02^a^ | 14.99±4.78^a^ | 0.570 |  |
| **Serum Creatinine (μmol/L)** | 95.05±35.80^a^ | 85.06±23.56^a^ | 0.020 | 0.100 |
| **Blood Urea Nitrogen (mmol/L)** | 6.02±1.85^a^ | 5.87±1.72^a^ | 0.570 |  |
| **Total Cholesterol (mmol/L)** | 4.30±0.77^a^ | 4.52±0.86^a^ | 0.060 |  |
| **Triglycerides (mmol/L)** | 1.39±0.95^a^ | 1.64±1.41^a^ | 0.130 |  |
| **Serum Low Density Lipoprotein Cholesterol (mmol/L)** | 2.44±0.51^a^ | 2.48±0.58^a^ | 0.580 |  |
| **Serum High Density Lipoprotein Cholesterol (mmol/L)** | 1.36±0.24^a^ | 1.29±1.34^a^ | 0.900 |  |
| **Heart Rate** | 71.45±10.75^a^ | 76.23±12.50^a^ | 0.004 | 0.030 |
| **His (μg/mL)** | 15.89±5.25^a^ | 15.75±4.96^a^ | 0.839 |  |
| **Hyp (μg/mL)** | 4.04±2.96^a^ | 3.18±2.07^a^ | 0.017 | 0.088 |
| **3MHis (μg/mL)** | 0.41±0.62^a^ | 0.32±0.41^a^ | 0.243 |  |
| **1MHis (μg/mL)** | 1.18±0.80^a^ | 0.78 0.45^a^ | 0.000 | 0.001 |
| **PEtN (μg/mL)** | 0.29±0.22^a^ | 0.22±0.16^a^ | 0.011 | 0.065 |
| **Asn (μg/mL)** | 7.15±1.79^a^ | 7.32±2.65^a^ | 0.590 |  |
| **Arg (μg/mL)** | 25.07±28.54^a^ | 15.90±7.00^a^ | 0.002 | 0.015 |
| **Car (μg/mL)** | 0.05±0.04^a^ | 0.09±0.05^a^ | 0.002 | 0.014 |
| **Tau (μg/mL)** | 20.39±6.83^a^ | 19.67±7.98^a^ | 0.491 |  |
| **Ans (μg/mL)** | 0.22±0.16^a^ | 0.22±0.16^a^ | 0.877 |  |
| **Ser (μg/mL)** | 16.66±3.96^a^ | 16.74±5.23^a^ | 0.906 |  |
| **Gln (μg/mL)** | 83.21±17.97^a^ | 78.12±22.82^a^ | 0.078 |  |
| **Asa (μg/mL)** | 0.08±0.16^a^ | 0.11±0.07^a^ | 0.160 |  |
| **Gly (μg/mL)** | 22.44±5.95^a^ | 23.33±7.96^a^ | 0.369 |  |
| **EtN (μg/mL)** | 0.66±0.26^a^ | 0.71±0.30^a^ | 0.206 |  |
| **Asp (μg/mL)** | 4.88±1.65^a^ | 5.24±2.01^a^ | 0.164 |  |
| **Cit (μg/mL)** | 7.87±3.04^a^ | 7.27±3.10^a^ | 0.170 |  |
| **Sar (μg/mL)** | 0.16±0.07^a^ | 0.14±0.07^a^ | 0.181 |  |
| **Glu (μg/mL)** | 18.55±8.79^a^ | 21.93±12.05^a^ | 0.023 | 0.105 |
| **bAla (μg/mL)** | 0.37±0.13^a^ | 0.35±0.15^a^ | 0.325 |  |
| **Thr (μg/mL)** | 16.26±4.46^a^ | 15.75±5.23^a^ | 0.456 |  |
| **Ala (μg/mL)** | 42.18±12.19^a^ | 43.52±15.67^a^ | 0.496 |  |
| **Hcit (μg/mL)** | 0.08±0.07^a^ | 0.08±0.05^a^ | 0.814 |  |
| **GABA (μg/mL)** | 0.03±0.02^a^ | 0.02±0.01^a^ | 0.005 | 0.028 |
| **Aad (μg/mL)** | 0.18±0.08^a^ | 0.17±0.07^a^ | 0.077 |  |
| **Hyl (μg/mL)** | 0.10±0.09^a^ | 0.05±0.05^a^ | 0.00 | 0.001 |
| **bAib (μg/mL)** | 0.31±0.27^a^ | 0.32±0.35^a^ | 0.88 |  |
| **Pro (μg/mL)** | 22.76±8.37^a^ | 22.11±8.33^a^ | 0.575 |  |
| **Cth (μg/mL)** | 0.16±0.20^a^ | 0.12±0.16^a^ | 0.199 |  |
| **Abu (μg/mL)** | 1.81±0.62^a^ | 1.91±0.85^a^ | 0.331 |  |
| **Cys (μg/mL)** | 14.70±4.40^a^ | 12.36±4.10^a^ | 0.000 | 0.001 |
| **Tyr (μg/mL)** | 13.22±3.59^a^ | 12.58±4.28^a^ | 0.246 |  |
| **Met (μg/mL)** | 3.98±1.02^a^ | 4.07±1.40^a^ | 0.601 |  |
| **Val (μg/mL)** | 30.10±8.01^a^ | 29.72±8.73^a^ | 0.746 |  |
| **Ile (μg/mL)** | 10.30±2.73^a^ | 9.67±3.10^a^ | 0.123 |  |
| **Leu (μg/mL)** | 20.65±4.95^a^ | 20.26±6.56^a^ | 0.636 |  |
| **Phe (μg/mL)** | 13.78±3.68^a^ | 13.98±4.57^a^ | 0.737 |  |
| **Trp (μg/mL)** | 11.68±2.92^a^ | 11.11±3.61^a^ | 0.214 |  |
| **Lys (μg/mL)** | 29.31±6.81^a^ | 29.17±8.56^a^ | 0.895 |  |

^a^ Values are presented as mean ± standard deviation

^#^ P value with Benjamin Hochberg correction

**Abbr.**: Histidine (His); 1-Methyl-L-histidine (1MHis); 3-Methyl-L-histidine (3MHis); Hydroxy- proline (Hyp); Asparagine (Asn); Phosphorylethanolamine (PEtN); Arginine (Arg); Carnosine (Car); Anserine (Ans); Argininosuccinic acid (Asa); Serine (Ser); Taurine (Tau); Glutamine (Gln); Ethanolamine (EtN); Glycine (Gly); Sarcosine (Sar); beta-Alanine (bAla); Threonine (Thr); Aspartic acid (Asp); Glutamic acid (Glu); Citrulline (Cit); Alanine (Ala); gamma-Aminobutyric acid (GABA); Aminoisobutyric acid (bAib); Proline (Pro); Aminoadipic acid (aAd); 5-Hydroxylysine (Hyl); Homocitrulline (Hcit); 2-Aminobutyric acid (Abu); Valine (Val); Methionine (Met); Tyrosine (Tyr); Cystathionine (Cth); Cystine (Cys); Leucine (Leu); Isoleucine (Ile); Phenylalanine (Phe); Tryptophan (Trp); Lysine (Lys)
