# Supplementary material for: The effect of fructose exposure on amino acid metabolism among Chinese community residents and its possible multi-omics mechanisms
Source: Sci Rep. 2023 Dec 19;13:22704. doi: 10.1038/s41598-023-50069-5 (PMC10733306; doi:10.1038/s41598-023-50069-5)
Supplement: Supplementary file 4 — Supplementary Table 4. [file 41598_2023_50069_MOESM4_ESM.docx]

**Supplementary table 4 Amino acid metabolic difference between high fructose exposure female population in the larger quartile and the accurately matched female control**

|  | **Control** | **High fructose concentration group** | **P value** | **Corrected P value^#^** |
| --- | --- | --- | --- | --- |
| **Mean/median of fructose (μg/mL)** | 0 | 469.78/318.35 |  |  |
| **The highest/lowest level of fructose (μg/mL)** | 0 | 1704.64/41.29 |  |  |
| **Age** | 68.66±10.24^a^ | 68.66±10.25^a^ | 1.000 |  |
| **Female** | 156 | 156 |  |  |
| **Diabetes (n)** | 30 | 32 |  |  |
| **Uric acid (μMol.L^-1^)** | 304.94±66.39^a^ | 304.63±72.32^a^ | 0.969 |  |
| **Height (cm)** | 153.50±6.50^a^ | 153.44 ±5.38^a^ | 0.937 |  |
| **Weight (Kg)** | 55.60±8.67^a^ | 54.41±8.54^a^ | 0.225 |  |
| **Waist Circumference (cm)** | 80.72±8.85^a^ | 78.56±10.16^a^ | 0.046 | 0.200 |
| **BMI (kg/cm2)** | 23.59±3.34^a^ | 23.07±3.19^a^ | 0.160 |  |
| **Left Systolic Blood Pressure (mm/Hg)** | 138.66±18.50^a^ | 138.77±19.14^a^ | 0.961 |  |
| **Left Diastolic Blood Pressure (mm/Hg)** | 73.46±7.65^a^ | 73.45±7.79^a^ | 0.994 |  |
| **Right Systolic Blood Pressure (mm/Hg)** | 142.12±18.53^a^ | 142.31±18.90^a^ | 0.932 |  |
| **Right Diastolic Blood Pressure (mm/Hg)** | 76.79±7.58^a^ | 77.11±7.73^a^ | 0.712 |  |
| **Hemoglobin (g/L)** | 126.78±11.60^a^ | 126.08±12.13^a^ | 0.607 |  |
| **White Blood Cells (×10**9/L) | 6.88±5.25^a^ | 6.44±1.62^a^ | 0.323 |  |
| **Platelet (×10**9/**L)** | 217.82±62.99^a^ | 217.78±63.81^a^ | 0.996 |  |
| **Fasting Blood Glucose (mmol/L)** | 6.28±1.55^a^ | 6.66±2.61^a^ | 0.127 |  |
| **Serum ALT (U/L)** | 21.94±15.14^a^ | 21.10±10.92^a^ | 0.578 |  |
| **Serum AST (U/L)** | 23.31±11.26^a^ | 22.38±7.88^a^ | 0.402 |  |
| **Total Bilirubin (μMol/L)** | 13.12±4.30^a^ | 12.55±4.30^a^ | 0.240 |  |
| **Serum Creatinine (μmol/L)** | 69.18±22.96^a^ | 65.99±15.26^a^ | 0.151 |  |
| **Blood Urea Nitrogen (mmol/L)** | 5.61±1.74^a^ | 5.44±1.35^a^ | 0.336 |  |
| **Total Cholesterol (mmol/L)** | 4.83±0.99^a^ | 4.92±1.03^a^ | 0.410 |  |
| **Triglycerides (mmol/L)** | 1.53±1.11^a^ | 1.93±1.71^a^ | 0.016 | 0.072 |
| **Serum Low Density Lipoprotein Cholesterol (mmol/L)** | 2.70±0.70^a^ | 2.67±0.73^a^ | 0.710 |  |
| **Serum High Density Lipoprotein Cholesterol (mmol/L)** | 1.57±0.32^a^ | 1.51±0.34^a^ | 0.079 |  |
| **Heart Rate** | 72.57±9.55^a^ | 73.65±11.35^a^ | 0.369 |  |
| **His (μg/mL)** | 15.29±4.67^a^ | 15.73±5.23^a^ | 0.434 |  |
| **Hyp (μg/mL)** | 3.15±2.18^a^ | 2.96±2.16^a^ | 0.427 |  |
| **3MHis (μg/mL)** | 0.48±1.13^a^ | 0.33±0.45^a^ | 0.110 |  |
| **1MHis (μg/mL)** | 0.97±0.78^a^ | 0.72±0.37^a^ | 0.001 | 0.007 |
| **PEtN (μg/mL)** | 0.31±0.20^a^ | 0.22±0.15^a^ | 0.000 | 0.000 |
| **Asn (μg/mL)** | 7.04±1.89^a^ | 7.17±2.40^a^ | 0.598 |  |
| **Arg (μg/mL)** | 22.53±20.89^a^ | 17.54±12.73^a^ | 0.012 | 0.062 |
| **Car (μg/mL)** | 0.08±0.05^a^ | 0.07±0.06^a^ | 0.871 |  |
| **Tau (μg/mL)** | 21.97±6.67^a^ | 22.37±8.10^a^ | 0.634 |  |
| **Ans (μg/mL)** | 0.17±0.15^a^ | 0.23±0.16^a^ | 0.001 | 0.012 |
| **Ser (μg/mL)** | 17.47±4.19^a^ | 17.40±5.92^a^ | 0.907 |  |
| **Gln (μg/mL)** | 82.76±21.55^a^ | 76.36±19.99^a^ | 0.007 | 0.040 |
| **Asa (μg/mL)** | 0.07±0.07^a^ | 0.10±0.05^a^ | 0.000 | 0.001 |
| **Gly (μg/mL)** | 25.43±9.26^a^ | 25.87±10.37^a^ | 0.692 |  |
| **EtN (μg/mL)** | 0.66±0.28^a^ | 0.77±0.35^a^ | 0.002 | 0.014 |
| **Asp (μg/mL)** | 5.16±1.85^a^ | 5.96±2.49^a^ | 0.001 | 0.014 |
| **Cit (μg/mL)** | 6.96±2.68^a^ | 6.70±2.30^a^ | 0.357 |  |
| **Sar (μg/mL)** | 0.14±0.07^a^ | 0.13±0.05^a^ | 0.087 |  |
| **Glu (μg/mL)** | 18.43±8.42^a^ | 21.69±10.93^a^ | 0.003 | 0.026 |
| **bAla (μg/mL)** | 0.32±0.15^a^ | 0.30±0.11^a^ | 0.212 |  |
| **Thr (μg/mL)** | 16.02±4.42^a^ | 15.38±4.91^a^ | 0.230 |  |
| **Ala (μg/mL)** | 43.89±13.03^a^ | 43.39±14.36^a^ | 0.749 |  |
| **Hcit (μg/mL)** | 0.08±0.06^a^ | 0.08±0.05^a^ | 0.432 |  |
| **GABA (μg/mL)** | 0.03±0.02^a^ | 0.02±0.01^a^ | 0.000 | 0.004 |
| **Aad (μg/mL)** | 0.16±0.07^a^ | 0.14±0.05^a^ | 0.005 | 0.033 |
| **Hyl (μg/mL)** | 0.11±0.10^a^ | 0.05±0.04^a^ | 0.000 | 1.90E-08 |
| **bAib (μg/mL)** | 0.25±0.25^a^ | 0.25±0.23^a^ | 0.995 |  |
| **Pro (μg/mL)** | 20.69±7.14^a^ | 21.22±8.10^a^ | 0.539 |  |
| **Cth (μg/mL)** | 0.12±0.12^a^ | 0.09±0.14^a^ | 0.209 |  |
| **Abu (μg/mL)** | 1.92±0.70^a^ | 1.81±0.72^a^ | 0.150 |  |
| **Cys (μg/mL)** | 14.72±6.43^a^ | 13.13±4.48^a^ | 0.012 | 0.058 |
| **Tyr (μg/mL)** | 13.50±3.81^a^ | 12.69±3.60^a^ | 0.052 |  |
| **Met (μg/mL)** | 3.76±1.02^a^ | 3.73±1.16^a^ | 0.795 |  |
| **Val (μg/mL)** | 28.66±7.00^a^ | 27.90±8.00^a^ | 0.372 |  |
| **Ile (μg/mL)** | 8.90±2.32^a^ | 8.67±2.73^a^ | 0.423 |  |
| **Leu (μg/mL)** | 18.69±4.56^a^ | 19.24±7.92^a^ | 0.460 |  |
| **Phe (μg/mL)** | 13.30±3.34^a^ | 14.02±4.21^a^ | 0.095 |  |
| **Trp (μg/mL)** | 10.98±2.81^a^ | 10.76±3.32^a^ | 0.517 |  |
| **Lys (μg/mL)** | 28.92±7.07^a^ | 28.98±7.49^a^ | 0.934 |  |

^a^ Values are presented as mean ± standard deviation

^#^ P value with Benjamin Hochberg correction

**Abbr.**: Histidine (His); 1-Methyl-L-histidine (1MHis); 3-Methyl-L-histidine (3MHis); Hydroxy- proline (Hyp); Asparagine (Asn); Phosphorylethanolamine (PEtN); Arginine (Arg); Carnosine (Car); Anserine (Ans); Argininosuccinic acid (Asa); Serine (Ser); Taurine (Tau); Glutamine (Gln); Ethanolamine (EtN); Glycine (Gly); Sarcosine (Sar); beta-Alanine (bAla); Threonine (Thr); Aspartic acid (Asp); Glutamic acid (Glu); Citrulline (Cit); Alanine (Ala); gamma-Aminobutyric acid (GABA); Aminoisobutyric acid (bAib); Proline (Pro); Aminoadipic acid (aAd); 5-Hydroxylysine (Hyl); Homocitrulline (Hcit); 2-Aminobutyric acid (Abu); Valine (Val); Methionine (Met); Tyrosine (Tyr); Cystathionine (Cth); Cystine (Cys); Leucine (Leu); Isoleucine (Ile); Phenylalanine (Phe); Tryptophan (Trp); Lysine (Lys)
